# Testing and Validating Green Dental Laboratory ‘Knowledge, Attitude, and Practice’ (KAP) Scale Developed for Dental Technicians Doing Prosthodontic Work

**DOI:** 10.3390/healthcare14203399

**Published:** 2026-10-12

**Authors:** Ayşegül Hazır, Elif Cil, Ömer Faruk Sönmez, Aysel Başer

**Affiliations:** 1 Dental Prosthesis Technology Program, Vocational School of Health Services, Kırıkkale University, 71450 Kırıkkale, Türkiye; aysegulhazir@gmail.com; 2 School of Medicine and Population Health, University of Sheffield, Sheffield S10 2TN, UK; 3 Karaman Provincial Health Directorate, 70100 Karaman, Türkiye; 4 Oral Health Program, Vocational School of Health Services, Kırıkkale University, 71450 Kırıkkale, Türkiye; omerfaruksnmez@gmail.com; 5 Department of Medical Education, Faculty of Medicine, İzmir Demokrasi University, 35140 İzmir, Türkiye; aysel.baser@idu.edu.tr; 6 Department of Biyoinformatik and Telemedicine, Jagiellonian University Medical College, 30-688 Krakow, Poland

**Keywords:** Green Dental Laboratory, sustainable dentistry, KAP model, scale validation

## Abstract

**Background:** Dental laboratories place a considerable burden on the environment through hazardous materials such as methyl methacrylate and gypsum, intensive energy and water use, waste generation, and transport between clinics and laboratories. Although Knowledge, Attitude and Practice (KAP) models are widely used to analyse professional behaviours, no standardised, psychometrically validated instrument exists for dental laboratory technicians. This study evaluated the preliminary psychometric properties of the newly developed “Green Dental Laboratory KAP Scale”. **Methods:** A methodological, cross-sectional scale development study was conducted with a convenience sample of 194 dental technology students and graduates. The 49-item instrument covered three domains: knowledge (11 items, formative), attitude (19 items, reflective), and practice (19 items, formative). The attitude construct was examined with Exploratory Factor Analysis (EFA) and Confirmatory Factor Analysis (CFA), and the formative constructs and structural relationships were examined with Partial Least Squares Structural Equation Modeling (PLS-SEM). **Results:** Most participants had never heard of the “Green Laboratory” concept (84.0%) and had not received formal environmental training (89.7%). The attitude scale showed a unidimensional structure (eigenvalue = 12.326; 82.17% of total variance) with high internal consistency (Cronbach’s α = 0.984, AVE = 0.807). CFA fit indices were mixed: CFI (0.917), TLI (0.903), and SRMR (0.030) met the recommended cut-offs. However, the robust RMSEA (0.130) exceeded the accepted cut-off. All formative indicators showed acceptable collinearity (VIF < 5). Knowledge was positively associated with both attitude (β = 0.308, *p* = 0.002) and practice (β = 0.553, *p* < 0.001), and attitude with practice (β = 0.362, *p* < 0.001), a pattern consistent with partial mediation. The structural model explained 56.0% of the variance in practice (R^2^ = 0.560). **Conclusions:** The Green Dental Laboratory KAP Scale is a preliminary psychometric evidence tool for assessing sustainability competencies in dental technology education and professional practice. The findings suggest that, alongside knowledge acquisition, cultivating pro-environmental attitudes is central to the adoption of green laboratory practices.

## 1. Background

The global healthcare sector accounts for a significant share of global greenhouse gas emissions, with dentistry contributing a large portion of this environmental burden [1,2]. The environmental footprint of healthcare services stems from factors such as energy consumption, water usage, the widespread use of single-use materials, and waste generation [3]. Although healthcare services primarily aim to protect and improve lives, they also contribute significantly to climate change. This situation creates an ethical paradox, making the transition to sustainable practices across all stages of the sector a necessity [3]. Green (or sustainable) dentistry practices aim to reduce the ecological impact of oral health services while maintaining high standards of care [4,5]. Green dentistry encompasses sustainable approaches such as energy-efficient technologies, digital dentistry, and eco-friendly sterilization techniques [6].

Prosthetic dentistry, among dental specialties, is one of the most carbon-intensive due to the numerous steps involved in producing a single product [7]. Every phase of production—impression handling, model fabrication, casting, finishing, and polishing—consumes large quantities of mostly disposable materials, such as polymers, ceramics, metals, gypsum, and wax, and generates its own waste stream [8]. Transport adds a further, often overlooked, burden: a recent carbon footprint study of private dental laboratories found that staff travel and the courier shipment of impressions and prostheses between clinics and laboratories accounted for the largest share of laboratory emissions (43.6%), followed by the procurement of materials over long supply chains and the energy consumed by furnaces, casting units, and milling equipment [8,9]. Part of the waste generated is also hazardous to health and the environment. Methyl methacrylate (MMA), the monomer of acrylic resins, is frequently used in prosthetic laboratories, and dentists and dental technicians exposed to MMA have reported hypersensitivity, asthma symptoms, local neurological symptoms, irritation, and dermatological reactions [10]. Moreover, disposing of gypsum waste without recycling increases solid waste generation, energy and water consumption, and waste management costs [11]. At the same time, innovation can act as an enabler of sustainability. Digital workflows reduce material waste and eliminate the need for multiple visits and temporary prostheses [12]. Although digital technologies raise new environmental issues of their own, such as energy consumption and contamination from waste particles generated during milling [13], life cycle assessments indicate that intraoral scanning combined with the use of local laboratories can substantially lower the carbon footprint of prosthesis fabrication, mainly by reducing material use and transport [14,15,16,17]. Integrating sustainability principles is therefore essential in dental prosthetics technology, which is characterized by significant laboratory waste, intensive resource use, and transport-dependent workflows.

The Knowledge, Attitude, and Practice (KAP) model is a widely used tool for understanding and changing health-related behaviors [18,19]. A KAP-based approach is particularly relevant to environmental topics because sustainability is, at its core, a matter of behaviour change: professionals must not only know which practices harm the environment, but must also believe that their individual actions can make a difference before they alter their daily routines [20,21]. The model assumes that an individual’s level of knowledge regarding a subject, their attitude toward it, and their actual behaviors in daily practice are interrelated; however, these relationships do not guarantee a direct causal link, and translating knowledge and attitudes into behavior often depends on other mediating variables [22,23]. In dentistry, KAP studies about infection control, biosafety, and waste management have shown significant gaps between knowledge and practice [24,25,26,27]. Methodologically, contemporary KAP instruments combine constructs of different natures: knowledge and practice items typically function as formative indicators that jointly define their construct, whereas attitude items are reflective expressions of a common underlying disposition, so their validation requires a combination of factor-analytic and structural equation modeling techniques [28,29]. One of the first systematic attempts to apply this mixed approach in green dentistry is a pilot psychometric study of a KAP questionnaire on eco-friendly dentistry, which also clearly highlighted the lack of standardized tools and the methodological heterogeneity that limits comparability across existing studies [28].

Most studies evaluating environmental awareness and green dentistry practices among healthcare professionals and dental students are cross-sectional and descriptive [30,31], and the existing research focuses almost exclusively on dental students or practicing dentists [30,32,33]. Dental laboratory technicians, by contrast, remain a largely overlooked group in dental research; a recent scoping review of occupational chemical exposures in dentistry, for example, found that technicians were investigated in only four of the thirty included studies and explicitly highlighted the lack of focus on this group [34]. This neglect is difficult to justify from a sustainability standpoint. Technicians are primarily responsible for decision-making regarding material selection, waste management, and energy use at every stage of dental prosthesis fabrication, and educating them in sustainable practices has been identified as a key strategy for mitigating the carbon emissions of dental laboratories [8]. To date, however, no psychometrically validated instrument has been developed to measure green dentistry KAP in this professional group.

This study therefore aimed to examine the psychometric properties of the “Green Dental Laboratory KAP Scale,” which was developed to assess the knowledge, attitudes, and practices regarding green dentistry among students and graduates of dental prosthesis technology programs, and to elucidate the structural relationships among these KAP dimensions. A psychometrically validated scale for this professional group could fill a significant gap in sustainability research and provide researchers and educators with a standardized, comparable measurement tool.

## 2. Methods

### 2.1. Study Design, Setting and Participants

This methodological, cross-sectional, scale-development study was conducted to evaluate the psychometric properties of the Green Dental Laboratory Knowledge–Attitude–Practice (KAP) Scale among dental laboratory technicians in Turkey. In Turkey, dental laboratory technicians are defined as individuals who fabricate dental prostheses and appliances under a dentist’s prescription without providing direct patient care. This qualification is obtained through a two-year associate degree in Dental Prosthesis Technology programs at vocational health schools; the scope of practice, training duration, and regulatory recognition of this role can differ substantially in other countries. Participants were either students enrolled in these programs with practical experience gained through internships, or graduate technicians already working in the field.

A convenience sampling strategy was employed among the students and graduates of Kırıkkale University, Vocational School of Health Services, which is appropriate for preliminary psychometric validation studies. Sample size adequacy was evaluated against established psychometric recommendations, which suggest a minimum of 5–10 participants per item and at least 190 participants for factor analysis [35]. Following data cleaning and the exclusion of duplicate and incomplete responses, the final sample consisted of 194 participants and was considered adequate for the planned psychometric analyses. Given the available sample size, splitting the dataset into separate exploratory and confirmatory subsamples would have substantially reduced the statistical power of both analyses. Therefore, EFA and CFA were conducted using the same sample. Accordingly, the CFA was treated as an internal and preliminary confirmation of the factor structure identified by EFA, rather than as an independent cross-validation.

### 2.2. Instrument Development

**Figure 1:**
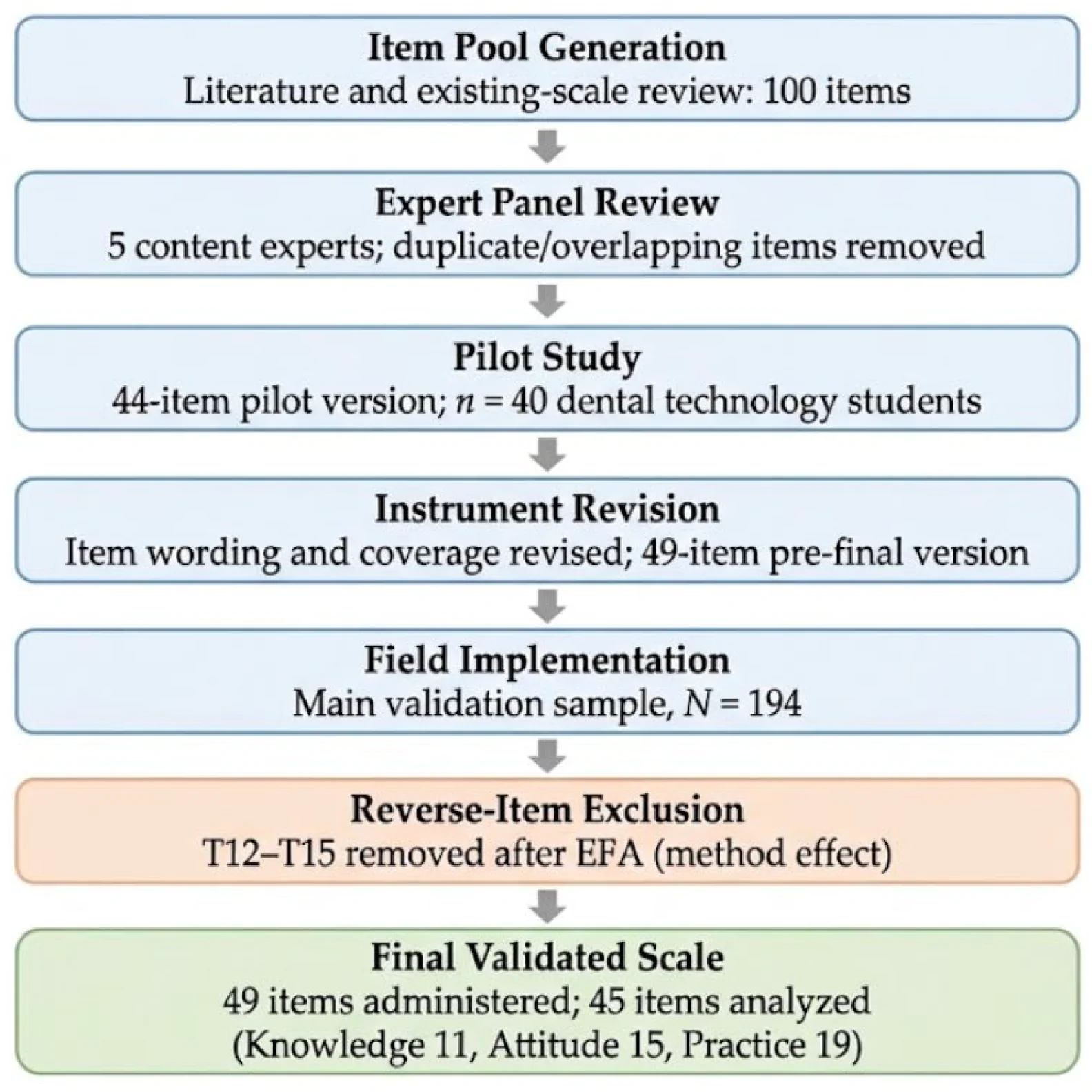
Development and refinement of the Green Dental Laboratory KAP Scale.

The Green Dental Laboratory KAP Scale was developed in three stages, following established guidance for scale development in health research [35] and informed by existing KAP instruments in green dentistry [28]. First, an initial pool of 100 items (25 knowledge, 35 attitude, and 40 practice items) was generated through a review of the green dentistry and sustainable healthcare literature. Second, this pool was evaluated by a panel of five content experts, comprising a professor of medical education, an academic in endodontics, and two academics in prosthodontics and dental technology, who assessed each item for relevance, clarity, and domain fit; items judged to be duplicative, ambiguous, or conceptually overlapping were removed, yielding a 44-item pilot instrument (11 knowledge, 14 attitude, and 19 practice items). Third, the pilot instrument was administered online to 40 dental technology students to evaluate item clarity and comprehensibility before the main data collection (Figure 1).

Pilot item statistics and expert feedback then guided the item-level revisions summarized in Table 1. In the knowledge domain, three items (B1, B3, B7) were reworded after showing poor discrimination and very high or very low difficulty in the pilot sample, while the remaining eight items were retained unchanged. In the attitude domain, only one pilot item (T16) survived unchanged; the other thirteen were reworded, largely to shift from a personal-desire framing toward a normative or benefit-based framing, and five new items (T7, T11, T14, T17, T19) were added to capture dimensions not represented in the pilot version, expanding the domain from 14 to 19 items and increasing the reverse-worded subset from three to four. In the practice domain, six items were retained unchanged, and thirteen were reworded, most often to shift from first-person statements about individual behaviour toward statements describing laboratory-level systems and infrastructure. These revisions produced the 49-item pre-final version administered to the main validation sample (N = 194). The scale was administered in Turkish; the final version, along with an English translation produced through translation and back-translation, is provided in the Supplementary Material. Researchers wishing to use or adapt the scale in other cultural or regulatory contexts should conduct further cross-cultural adaptation and validation.

### 2.3. Instrument Structure and Data Management

The questionnaire administered to participants consisted of 49 items distributed across three domains: knowledge (11 items), attitude (19 items), and practice (19 items) (Table 2). Consistent with contemporary KAP methodology, we treated the knowledge and practice constructs as formative, while the attitude construct was reflective [19,20]. Data were prepared by removing duplicate records and coding variables before analysis. During preliminary analysis, the four negatively worded attitude items (T12–T15) loaded onto a separate factor from the remaining attitude items and displayed markedly lower corrected item–total correlations (Supplementary Table S1), a pattern consistent with the method effect commonly reported for reverse-worded items embedded within otherwise positively worded scales [36]. We therefore excluded these four items from the factor-analytic and reliability analyses. Accordingly, although the administered questionnaire contained 49 items, the validated analytical core comprised 45 items: 11 knowledge items (B1–B11), 15 attitude items (T1–T11 and T16–T19), and 19 practice items (U1–U19). T12–T15 were not included in the validated Attitude score or its psychometric interpretation (Table 2). Several procedures were adopted to reduce potential sources of bias: data were collected anonymously and voluntarily to limit socially desirable responding, duplicate responses were identified and removed, reverse-worded items were included in the administered instrument to limit acquiescent responding [36], and the item pool was refined through independent expert review and pilot testing to reduce ambiguity-related measurement error.

Scale Scoring: Knowledge, Attitude, and Practice were scored as separate domains, and no overall KAP score was calculated due to the mixed formative-reflective structure. Knowledge items (B1–B11) were scored dichotomously (0/1) and summed to obtain a total score ranging from 0 to 11. The Attitude score was based on the 15 retained items (T1–T11 and T16–T19), excluding T12–T15, while the Practice score included all 19 items (U1–U19). Attitude and Practice items were scored on their original 1–5 response metric, and total domain scores were calculated (a maximum possible score of 75 for Attitude and 95 for Practice). Further details of the scoring procedure are provided in the Supplementary Material.

### 2.4. Statistical Analysis

Statistical analyses were conducted according to the measurement characteristics of each domain and the psychometric properties being evaluated.

Missing data were limited to a small number of Knowledge items (1–6 missing responses per item); no item-level missing data were observed for the Attitude or Practice items. No missing values were imputed. EFA used listwise deletion; because the retained Attitude items contained no missing values, all 194 participants were included in the analysis.

Exploratory factor analysis (EFA) was used to examine the underlying structure of the attitude domain and determine its dimensionality, and confirmatory factor analysis (CFA) was used to assess the validity of the reflective attitude construct. Partial least squares structural equation modeling (PLS-SEM) was employed to evaluate the formative knowledge and practice constructs and the structural relationships among the three domains, as conventional reliability indices such as Cronbach’s alpha are not appropriate for formative measurement models [29,37].

The Principal Axis Factoring (PAF) method was chosen for the EFA, as it is appropriate for identifying latent constructs when the data do not follow a normal distribution [38]. The number of factors to retain was determined using multiple criteria, including eigenvalues greater than 1 (Kaiser criterion) and visual inspection of the scree plot. A factor-loading threshold of 0.40 was adopted as the minimum acceptable level for item retention. Before EFA, the suitability of the data for factor analysis was evaluated with the Kaiser–Meyer–Olkin (KMO) measure of sampling adequacy and Bartlett’s Test of Sphericity.

Confirmatory factor analysis (CFA) was performed to assess the measurement model’s validity using the lavaan package in R, employing the robust maximum likelihood estimator (MLR) to account for potential nonnormality. Model fit was assessed using multiple goodness-of-fit indices, including the chi-square statistic and its ratio to degrees of freedom (χ^2^/df), the Comparative Fit Index (CFI), Tucker–Lewis Index (TLI), Root Mean Square Error of Approximation (RMSEA), and Standardized Root Mean Square Residual (SRMR). Model fit indices were interpreted using commonly recommended reference thresholds (CFI and TLI ≥ 0.90, SRMR ≤ 0.08, and RMSEA ≤ 0.08; RMSEA values > 0.10 were considered indicative of misfit) [35].

The knowledge and practice constructs, conceptualized as formative, were analyzed using PLS-SEM with the seminr package in R, mainly because of its suitability for formative measurement models and its robustness in prediction-oriented research [28,30]. The structural model was evaluated by examining path coefficients and their statistical significance using bootstrapping with 1000 resamples, and the model’s explanatory power was assessed with R^2^ values. The measurement model was evaluated by examining indicator outer loadings and outer weights. For the formative constructs, indicators were evaluated based on the statistical significance of their outer weights, outer loadings (threshold > 0.50), and theoretical relevance, following the decision rules recommended for formative measurement models [37,39], so that each item contributed meaningfully to the construct’s definition. Construct distinctiveness among the KAP domains (a key aspect of measurement model evaluation) was assessed using the inter-construct Pearson correlation matrix (Supplementary Table S5). Collinearity was assessed using the Variance Inflation Factor (VIF), with values below 5.0 indicating the absence of severe multicollinearity (Supplementary Table S4). All analyses were conducted using IBM SPSS Statistics version 32 and RStudio 2026.06.0+242, and the significance level was set at *p* < 0.05.

The methodological quality of the study was self-assessed against the COSMIN standards for evaluating measurement properties [40,41,42], and the completed assessment tables are provided in the Supplementary Material.

### 2.5. Ethical Considerations

The study was conducted in accordance with the Declaration of Helsinki and approved by the Non-Interventional Research Ethics Committee of Kırıkkale University on 16/04/2026 with decision number 2026.04.19. Participation was voluntary, and all participants provided informed consent before data collection. Participants were informed about the study purpose, confidentiality, and their right to withdraw at any time. Data were collected anonymously and used solely for scientific purposes.

## 3. Results

Most participants were female (59.8%) and aged 18–24 years (78.9%). The most common degree was an associate degree (73.7%), and more than half of the respondents had less than one year of professional experience (67.0%). The majority worked in private laboratories (69.1%) and had small teams of 1–5 personnel (57.7%). Notably, a large proportion of participants had not previously heard of green dentistry concepts (84.0%) and had not received formal sustainability training (89.7%) (Table 3). Item-level descriptive statistics for all 49 items, including corrected item–total correlations, are presented in Supplementary Table S1.

### 3.1. Exploratory Factor Analysis (Attitude Construct)

Before EFA, the Kaiser–Meyer–Olkin (KMO) measure of sampling adequacy was 0.967, exceeding the recommended threshold of 0.80, and Bartlett’s Test of Sphericity was statistically significant (χ^2^ = 4391.501, df = 105, *p* < 0.001), confirming that the correlation matrix was appropriate for factor analysis.

**Figure 2:**
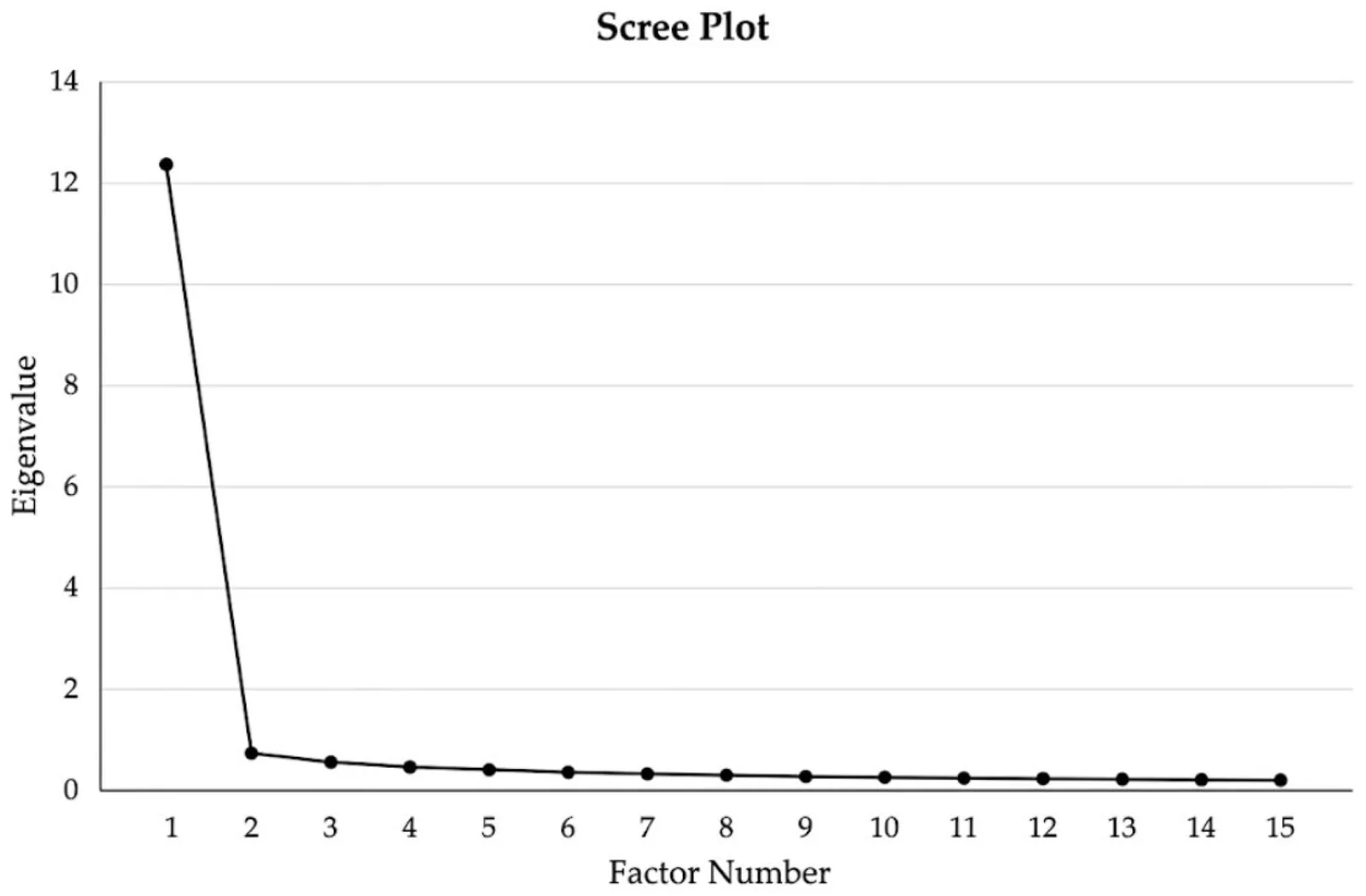
Scree plot shows the eigenvalues for one factor.

The number of factors was determined using eigenvalues greater than 1 (Kaiser criterion) and inspection of the scree plot. The results indicated a dominant single-factor structure, with the first factor having an eigenvalue of 12.326 and accounting for 82.17% of the total variance. The scree plot further supported a unidimensional solution, showing a clear inflection point after the first factor (Figure 2).

The Principal Axis Factoring solution showed factor loadings ranging from 0.835 to 0.934 and communalities from 0.698 to 0.872; the item-level loadings are reported in Supplementary Table S2.

### 3.2. Confirmatory Factor Analysis (CFA) (Attitude Construct)

The CFA provided preliminary internal support for the unidimensional structure of the attitude construct, with all 15 retained items (T1–T11, T16–T19) loading on a single latent factor. Standardized factor loadings ranged from 0.832 to 0.931 (all *p* < 0.001), exceeding the recommended threshold of 0.50; the full loading matrix is reported in Supplementary Table S3.

The model yielded a chi-square value of χ^2^(90) = 459.851 (*p* < 0.001; χ^2^/df = 5.109). The Comparative Fit Index (CFI) was 0.917, the Tucker–Lewis Index (TLI) was 0.903, and the Standardized Root Mean Square Residual (SRMR) was 0.030. In contrast, the uncorrected ML-based RMSEA was elevated to 0.146 (90% CI of ML-based RMSEA: 0.133–0.159), and the robust RMSEA was 0.130, exceeding the standard threshold for fit (RMSEA ≤ 0.08). Overall, the CFA results showed mixed fit indices: the CFI, TLI, and SRMR were within commonly accepted ranges, whereas the RMSEA values indicated misfit and a high level of discrepancy (Supplementary Table S3). This is most likely due to a single-factor model with high factor loadings; however, this does not mitigate the observed discrepancy [43].

### 3.3. PLS-SEM Results

**Figure 3:**
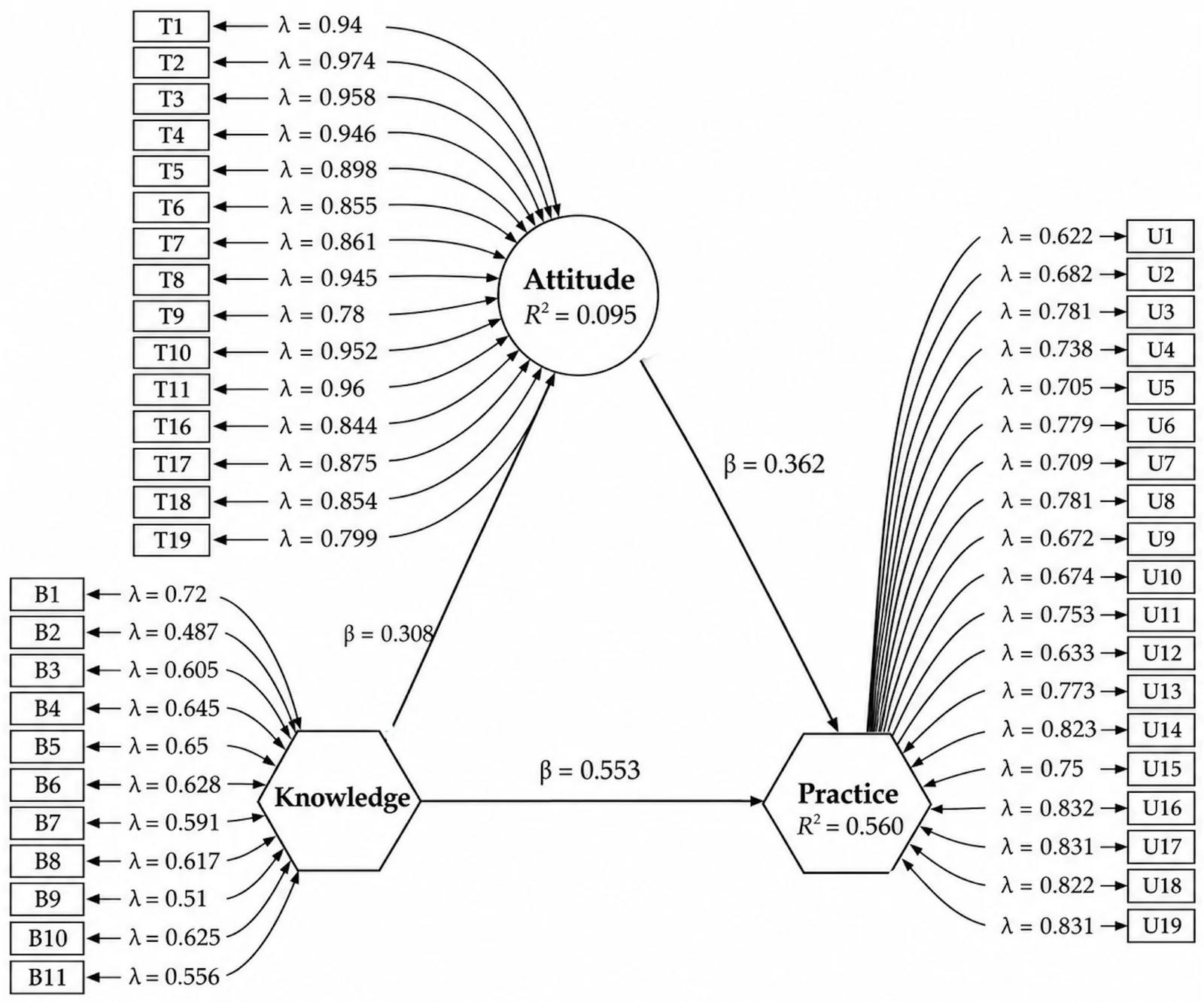
Structural equation model illustrating the relationships among knowledge, attitude, and practice constructs. Knowledge and practice were modelled as formative constructs, while attitude was specified as a reflective construct. Arrows pointing from or to indicators represent outer loadings (λ), while single-headed arrows between constructs represent path coefficients (β).

The structural model showed theoretically consistent relationships among the latent constructs (Figure 3). Bootstrapping results (n = 1000) indicated that all hypothesized paths were statistically significant. Knowledge was positively associated with attitude (β = 0.308, *p* = 0.002, 95% CI [0.115–0.495]) and, more strongly, with practice (direct effect: β = 0.553, *p* < 0.001, 95% CI [0.408–0.685]). Attitude was also positively associated with practice (β = 0.362, *p* < 0.001, 95% CI [0.192–0.521]).

**Figure 4:**
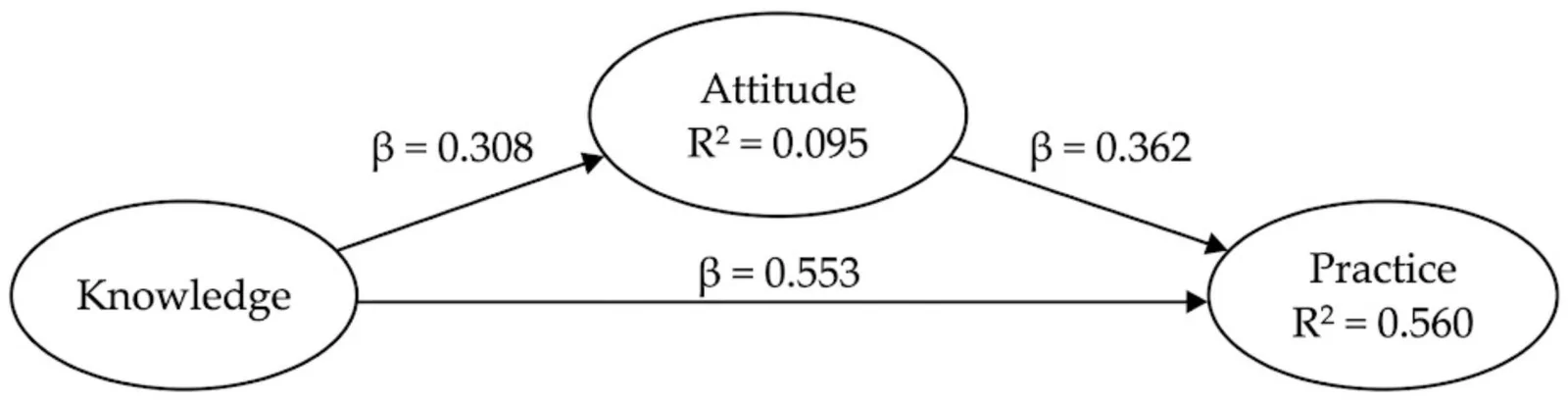
Minimal structural model of the Green Dental Laboratory KAP Scale. Standardized path coefficients are shown on the arrows. R^2^ values are displayed within endogenous constructs.

Formal mediation analysis confirmed that attitude significantly mediated the relationship between knowledge and practice, with a statistically significant indirect effect (β = 0.111, *p* = 0.002, 95% CI [0.048–0.188]). The total effect of knowledge on practice was also significant (β = 0.664, *p* < 0.001, 95% CI [0.531–0.776]), confirming a partial mediation model (Table 4). The model explained 9.5% of the variance in attitude (R^2^ = 0.095) and 56.0% of the variance in practice (R^2^ = 0.560) (Figure 4).

### 3.4. Measurement Model Assessment

The reflective attitude construct showed high internal consistency and convergent validity (Cronbach’s α = 0.984, composite reliability = 0.984, AVE = 0.807). For the formative constructs, either indicators had statistically significant outer weights (*p* < 0.05), or those indicators with non-significant outer weights were retained based on their theoretical relevance. Outer loadings ranged from 0.48 to 0.72 for the knowledge indicators and from 0.62 to 0.83 for the practice indicators (Supplementary Table S6).

### 3.5. Construct Distinctiveness

The inter-construct correlation matrix revealed no evidence of construct similarity (Supplementary Table S5). VIF values ranged from 1.230 to 1.721 for the knowledge construct and from 1.909 to 4.663 for the practice construct; all values remained below the conservative threshold of 5, indicating no problematic multicollinearity among the formative indicators (Supplementary Table S4).

## 4. Discussion

This study examined the psychometric properties of the Green Dental Laboratory KAP Scale, a tool developed to assess knowledge, attitudes, and practices regarding green dentistry among students and graduates in dental prosthetics technology, and elucidated the structural relationships among these KAP dimensions. The findings indicate that the scale exhibits good reliability and validity and provides a standardized tool for sustainability research within this professional group.

### 4.1. Psychometric Validation of the Scale

KAP research in green dentistry has long suffered from a methodological gap: most measurement tools have been applied without adequate psychometric evaluation, and the absence of standardized instruments limits comparability across studies [28,31]. The present study addresses this gap by combining EFA, CFA, and PLS-SEM within a single validation design, which allows the reflective and formative parts of the instrument to be evaluated with techniques appropriate to their measurement logic rather than through partial validity analyses alone.

Sampling adequacy for the attitude items was excellent (KMO = 0.967), and the large chi-square value of Bartlett’s test (χ^2^ = 4391.501) points to a strongly interrelated set of items. In practical terms, this means that participants who endorsed one pro-environmental attitude statement tended to endorse the others as well, which is a precondition for treating the attitude items as reflective indicators of a common underlying disposition.

A key result of this study is that the attitude structure appeared to be unidimensional, with the first factor explaining 82.17% of the total variance, even though five conceptual sub-domains had been considered during item development. This suggests that, in this population, evaluations of green laboratory practice are organized around a single, general pro-environmental disposition rather than differentiated sub-attitudes. One plausible explanation lies in the profile of the sample: most participants were young, at an early career stage, and largely unexposed to sustainability training, and attitudes tend to remain globally organized when respondents have limited direct experience with the attitude object. Multidimensional attitude structures reported for instruments developed in heterogeneous groups of dental students and staff [28] may therefore reflect both differences in item content and the greater professional diversity of those samples.

### 4.2. Structural Relationships Among Knowledge, Attitude, and Practice

The structural model findings are consistent with a central role of knowledge in sustainable action. PLS-SEM analyses showed that higher knowledge was associated with more positive attitudes (β = 0.308) and, more strongly, with better-reported practices (β = 0.553), in line with the literature identifying knowledge as a fundamental prerequisite for green dentistry [32]. Attitude was also directly associated with Practice (β = 0.362). Furthermore, Knowledge exerted a statistically significant indirect effect on Practice through Attitude (β = 0.111). This pattern confirms a partial mediation model, indicating that while attitude serves as a key mechanism translating knowledge into practice, knowledge also retains a strong direct influence on behavioural implementation. This also supports the theoretical sequence proposed by the KAP framework and by the pro-environmental behaviour literature, in which beliefs about the value of individual action link what professionals know to what they actually do [20]. Given that technician students are the future primary decisionmakers regarding material selection, waste management, and energy use at every stage of dental prosthesis production, these findings imply that curricula should aim not only at transferring knowledge but also at cultivating attitudes that internalize it.

The proportion of variance explained in practice (R^2^ = 0.560) indicates moderate-to-high explanatory power, whereas the low variance explained in attitude (R^2^ = 0.095) suggests that attitude formation is not primarily driven by knowledge but is also shaped by factors outside the model, such as experience, social norms, peer influence, and organizational culture. This is consistent with Bandura’s Social Cognitive Theory, in which changes in beliefs and attitudes emerge through social learning, role modelling, and observational experience rather than knowledge accumulation alone [44], and with the well-documented gap between environmental awareness and pro-environmental behaviour [18]. Identifying these additional determinants of attitude in dental laboratory settings is an important direction for future research.

### 4.3. Formative Constructs: Knowledge and Practice

Modelling the knowledge and practice constructs formatively is consistent with the theoretical structure of green dentistry instruments, in which the items within these domains are not interchangeable and each contributes a distinct facet of the construct [29,37]. Accordingly, the collinearity diagnostics indicated that all VIF values for both the knowledge (1.230–1.721) and practice (1.909–4.663) constructs remained below 5, confirming that each item makes a unique contribution to its construct without problematic redundancy.

Outer loading values for the knowledge construct ranged between 0.48 and 0.72. Although the loadings of some items fell slightly below the 0.50 threshold, these items were retained because of their theoretical contribution to the thematic areas, consistent with the decision-making process recommended by Hair et al. for formative models within the PLS-SEM framework [39] and with retention decisions reported in earlier formative KAP work in green dentistry [28]. The higher and more consistent outer loadings of the practice items (0.62–0.83) indicate that the practice indicators represent their construct more strongly.

### 4.4. Reflective Construct: Attitude

The attitude construct exhibited high internal consistency and convergent validity, with Cronbach’s α (0.984), composite reliability (0.984), and AVE (0.807) all exceeding the recommended thresholds for reflective measurement models [39]. At the same time, alpha values of this magnitude may indicate a degree of content overlap among items, since coefficients above 0.95 can signal item redundancy rather than purely superior measurement. Future revisions of the scale could therefore examine whether a shorter attitude item set preserves the breadth of the construct while reducing respondent burden.

### 4.5. Reverse-Worded Items and Method Effects

An informative by-product of the validation process concerns the four reverse-worded attitude items (T12–T15). As shown in Supplementary Table S1, these items displayed markedly lower corrected item–total correlations (0.109–0.438) than the positively worded attitude items (0.816–0.899) and loaded onto a separate factor, a pattern typical of the method variance introduced by negative wording rather than of a substantive sub-dimension [36]. Their exclusion clarified the factor structure, but it also narrowed the content coverage of the scale, as these items addressed the perceived financial and practical burdens of green practice. Rather than reintroducing them as reversed attitude statements, future versions of the scale could operationalize perceived barriers as a separate construct, allowing the economic and feasibility concerns reported in the green dentistry literature to be measured without distorting the attitude factor.

### 4.6. Comparison with Green Dentistry Research

The current findings are consistent with previous studies on green dentistry, indicating that knowledge is a fundamental correlate of sustainable behaviour [30,45,46,47]. However, unlike earlier research that assessed awareness primarily through descriptive or partially validated questionnaires, this study presents a psychometrically validated measurement tool developed specifically for early career dental technicians (dental prosthesis technology students). The structural model extends previous findings by showing that knowledge is related to sustainable practices both directly and indirectly through attitudes, thereby providing empirical support for the KAP model within the context of green dentistry.

Participants reported very low prior awareness of the “green laboratory” concept (84.0% unaware) and little previous sustainability training (89.7% untrained); these figures align with comprehensive reviews of environmental sustainability content in dental education [30,31]. Furthermore, knowledge explained only a small share of the variance in attitudes (R^2^ = 0.095), whereas its association with practices was much stronger, both directly (β = 0.553) and indirectly through attitudes (β = 0.362). While this mirrors the “awareness–practice” gap reported in earlier studies, those studies identified financial and infrastructural constraints, rather than a lack of knowledge, as the primary barriers to translating awareness into sustainable practice [48,49,50,51]. In our study, the high proportion of participants unfamiliar with the subject may also have introduced guessing or floor effects in the dichotomous knowledge items, so the observed associations may be under- or overestimated. Since perceived structural or financial barriers were not included in the current measurement tool, incorporating barrier-related items into future revisions of the scale would help future research address the knowledge–practice gap observed in the green dentistry literature.

### 4.7. Implications for Education and Practice

This study addresses a significant gap by focusing on dental technology, a professional group that has received limited attention despite the substantial environmental impact of prosthetic dentistry. This scale offers a standardized tool for assessing sustainability competencies, identifying educational needs, and measuring the effectiveness of green dentistry training programs. It may also facilitate comparisons between institutions and support the integration of sustainability into dental technology curricula.

### 4.8. Strengths and Limitations

This study has notable strengths, including the combined use of EFA, CFA, and PLS-SEM within a single validation design, the transparent reporting of item-level statistics and measurement diagnostics in the Supplementary Material, and its focus on dental laboratory technicians, a professional group rarely examined in the green dentistry literature.

Nevertheless, several limitations should be acknowledged. First, the cross-sectional design precludes any causal interpretation of the relationships among knowledge, attitude, and practice; the reported paths describe associations, and their direction remains theoretical. Second, participants were recruited by convenience sampling from a single institution’s dental prosthesis technology and oral health programs, and most (67.0%) had less than one year of professional experience; the sample may therefore better represent trainees than technicians with established decision-making authority in commercial laboratories, and selection bias cannot be excluded.

Third, all three KAP domains were assessed via self-report collected from the same source at the same time, which raises the possibility of social desirability and common method bias, particularly for the practice items [52,53]. Fourth, the knowledge domain was scored dichotomously; given the limited prior exposure of many participants to green dentistry terminology, this format is susceptible to guessing and may over- or underestimate actual knowledge.

Fifth, several limitations related to content validity should be noted under COSMIN standards. Item retention decisions relied on qualitative expert consensus and pilot statistics rather than a formalized quantitative Content Validity Index; target participants did not formally rate the comprehensiveness of the instrument; and the five-member academic panel did not include actively practicing laboratory technicians or environmental-health experts. In addition, excluding the four reverse-worded items (T12–T15) removed content addressing perceived economic and practical burdens, narrowing the attitude domain’s content coverage.

Sixth, structural validity requires further confirmation. The CFA of the attitude construct yielded an RMSEA above conventional cut-offs, indicating that model fit was not uniformly satisfactory across fit indices, which is a clear limitation for the study. Moreover, EFA and CFA were conducted using the same sample (N = 194), because splitting the available sample into separate exploratory and confirmatory subsamples would have substantially reduced the statistical power of both analyses. Consequently, the CFA does not constitute an independent cross-validation of the factor structure and should be regarded as providing preliminary internal evidence only. The proposed unidimensional structure of the attitude construct therefore requires confirmation in an independent sample. Finally, test–retest reliability and criterion validity against observed laboratory behavior were not assessed; the scale’s stability, responsiveness, and cross-cultural performance should therefore be examined in independent, multi-institutional samples.

## 5. Conclusions

This study provides psychometric evidence supporting a three-domain KAP instrument developed specifically for dental prosthesis technology students and technicians. The attitude scale exhibited a unidimensional factor structure with high reliability, while the knowledge and practice domains functioned as formative constructs, with each indicator contributing uniquely to its construct without problematic multicollinearity.

In summary, the Green Dental Laboratory Knowledge, Attitude, and Practice Scale provides a preliminary standardized framework for assessing and monitoring sustainability-related knowledge, attitudes, and practices among dental laboratory technicians and for evaluating the impact of curriculum- or workplace-based interventions designed to strengthen green dental laboratory practices. Future research should extend this validation to independent, multi-institutional, and international samples and incorporate longitudinal or intervention designs to examine whether enhancing knowledge and attitudes is followed by measurable, lasting changes in laboratory practice over time.

## Figures and Tables

**Table 1:** Item-Level Disposition from Pilot (44 items) to Pre-Final Version (49 items).

Domain	Retained Unchanged	Revised (Reworded)	Added
Knowledge (B)	B2, B4, B5, B6, B8, B9, B10, B11	B1, B3, B7	—
Attitude (T)	T16	T1, T2, T3, T4, T5, T6, T8, T9, T10, T12, T13, T15, T18	T7, T11, T14, T17, T19
Practice (U)	U2, U4, U5, U7, U12, U17	U1, U3, U6, U8, U9, U10, U11, U13, U14, U15, U16, U18, U19	—

**Table 2:** Structure of the validated analytical core of the Green Dental Laboratory KAP Scale.

Section (Construct)	Items	Response Format	Measurement Model	Analysis Method
Knowledge	B1–B11 (11 items)	Dichotomous (Yes = 1/No = 0)	Formative	PLS-SEM
Attitude	T1–T11, T16–T19 (15 retained items) *	5-point Likert scale (1 = Strongly Disagree to 5 = Strongly Agree)	Reflective	EFA and CFA
Practice	U1–U19 (19 items)	5-point Likert scale (1 = Never to 5 = Always)	Formative	PLS-SEM

* The questionnaire administered to participants contained 49 items. Four reverse-worded attitude items (T12–T15) were excluded during psychometric evaluation; therefore, the validated analytical core comprised 45 items. T12–T15 were not included in the validated Attitude score or subsequent psychometric interpretation.

**Table 3:** Descriptive Characteristics of the Participants.

Variables		(n)	(%)
Gender	Women	116	59.8
	Men	78	40.2
Age (years)	18–24	153	78.9
	25–34	24	12.4
	35–44	12	6.2
	45–55	4	2.1
	55+	1	0.5
Education Level	High school	33	17.0
	Associate degree	143	73.7
	Bachelor’s degree	17	8.8
	Postgraduate	1	0.5
Professional Experience	<1 year	130	67.0
	1–5 years	41	21.1
	6–10 years	13	6.7
	11–20 years	4	2.1
	>20 years	6	3.1
Type of Laboratory	Private	134	69.1
	Public/Hospital	29	14.9
	University	29	14.9
	Other	2	1.0
Number of Laboratory Staff	1–5	112	57.7
(persons)	6–10	48	24.7
	11–20	18	9.3
	>20	16	8.2
Prior Awareness of the	No	163	84.0
“Green Laboratory” Concept	Yes	31	16.0
Prior Receipt of Environmental	No	174	89.7
Sustainability Training	Yes	20	10.3

**Table 4:** Direct, Indirect, and Total Effects for the Mediation Model.

Direction	Std. Est. (β)	BM	BSE	t-Value	95% BCI
Knowledge → Attitude (a)	0.308	0.309	0.098	3.143	0.115–0.495
Attitude →* Practice (b)	0.362	0.363	0.085	4.258	0.192–0.521
Knowledge →* Practice (c)	0.553	0.552	0.071	7.788	0.408–0.685
Indirect effect (a × b)	0.111	0.112	0.035	3.171	0.048–0.188
Total effect	0.664	0.664	0.063	10.540	0.531–0.776

* Bootstrap Mean: BM, Bootstrap SE: BSE, Bootstrapped CI: BCI.

## Data Availability

The datasets analyzed during the current study are available from the Mendeley data repository through https://doi.org/10.17632/3z2xtv83kv.1.

## Supplementary Materials

The following supporting information can be downloaded at: https://www.mdpi.com/article/10.3390/healthcare14203399/s1, Table S1. Item-Level Descriptive Statistics (N = 194), Table S2. Exploratory Factor Analysis of the Attitude Construct, Table S3. Confirmatory Factor Analysis of the Attitude Construct, Table S4. Formative Indicator Collinearity Statistics (VIF), Table S5. Cross Loadings/Correlation Matrix, Table S6. Formative Indicator Measurement Model Assessment, Green Dental Laboratory Knowledge, Attitude, and Practice (KAP) Scale and COSMIN Assessment.

## References

[1] Lenzen M., Malik A., Li M., Fry J., Weisz H., Pichler P.P., Chaves L.S.M., Capon A., Pencheon D. (2020). The environmental footprint of health care: A global assessment. Lancet Planet. Health.

[2] Romanello M., Napoli C.D., Green C., Kennard H., Lampard P., Scamman D., Walawender M., Ali Z., Ameli N., Ayeb-Karlsson S. (2023). The 2023 report of the Lancet Countdown on health and climate change: The imperative for a health-centred response in a world facing irreversible harms. Lancet.

[3] MacNeill A.J., Hopf H., Khanuja A., Alizamir S., Bilec M., Eckelman M.J., Hernandez L., McGain F., Simonsen K., Thiel C. (2020). Transforming The Medical Device Industry: Road Map To A Circular Economy: Study examines a medical device industry transformation. Health Aff..

[4] Duane B., Steinbach I., Ramasubbu D., Stancliffe R., Croasdale K., Harford S., Lomax R. (2019). Environmental sustainability and travel within the dental practice. Br. Dent. J..

[5] Martin N., Dixon J., Mulligan S. (2025). Status update: Environmental sustainability in the dental profession. Br. Dent. J..

[6] Saxena G., Rathore K., Kumar V. (2025). Green Dentistry: A Sustainable Approach to Modern Dental Practice. Indian J. Dent. Sci..

[7] Shinkai R.S.A., Biazevic M.G.H., Michel-Crosato E., De Campos T.T. (2025). Environmental sustainability related to dental materials and procedures in prosthodontics: A critical review. J. Prosthet. Dent..

[8] Elwan A.H., Fouda A.M. (2025). Carbon footprint of private dental laboratories in Egypt: A cross-sectional study. BDJ Open.

[9] Martin N., Sheppard M., Gorasia G., Arora P., Cooper M., Mulligan S. (2021). Drivers, opportunities and best practice for sustainability in dentistry: A scoping review. J. Dent..

[10] Leggat P.A., Kedjarune U. (2003). Toxicity of methyl methacrylate in dentistry. Int. Dent. J..

[11] Tawade S.U., Dhamande M.M., Dubey S.A., Sathe S., Selukar M.S., Pathak A. (2024). Sustainable Dentistry: A Comprehensive Review of the Recycling Techniques for Gypsum Products in Prosthodontics. Cureus.

[12] Khurshid Z. (2023). Digital Dentistry: Transformation of Oral Health and Dental Education with Technology. Eur. J. Dent..

[13] Guerra M., Morgado M., Leira Y., Leitão T., Botelho J., Mendes J.J. (2025). Integrating sustainability in dentistry: A pathway towards achieving the UN 2030 agenda. Front. Oral Health.

[14] Rosenblatt A., Sabu P., McAuley K., Ryan E., Malaj T., Tiew N., Sheet A., Zheng J., Corr S., Duane B. (2026). Life cycle assessment of the complete denture fabrication process using traditional impressions versus intra-oral scanning. Br. Dent. J..

[15] Sahni J., Lin W., Kilinc E., Abdelaal M., Ganesan S., Spears R., Karl E. (2024). Advancing climate change education in the dental curriculum. J. Dent. Educ..

[16] Licari F.W., Patil S. (2024). Climate change and the unforeseen challenges for dental practice. J. Am. Dent. Assoc..

[17] Frumkin H., Hess J., Luber G., Malilay J., McGeehin M. (2008). Climate Change: The Public Health Response. Am. J. Public Health.

[18] Zarei F., Dehghani A., Ratansiri A., Ghaffari M., Raina S., Halimi A., Rakhshanderou S., Ismael S.A., Amiri P., Aminafshar A. (2024). ChecKAP: A Checklist for Reporting a Knowledge, Attitude, and Practice (KAP) Study. Asian Pac. J. Cancer Prev..

[19] Himanshu Balkumar G., Mutha R., BM, Akash P., Vikram K., Ritik K. (2025). A KAP-based evaluation on the role of climate change in shaping dental practices. Bioinformation.

[20] Kollmuss A., Agyeman J. (2002). Mind the Gap: Why do people act environmentally and what are the barriers to pro-environmental behavior?. Environ. Educ. Res..

[21] Dixon J., Martin N., Field J. (2024). Current practice, barriers and drivers to embedding environmental sustainability in undergraduate dental schools in the UK and Ireland. Br. Dent. J..

[22] Wang Q., Wu Y., Wang D., Lai X., Tan L., Zhou Q., Duan L., Lin R., Wang X., Zheng F. (2023). The impacts of knowledge and attitude on behavior of antibiotic use for the common cold among the public and identifying the critical behavioral stage: Based on an expanding KAP model. BMC Public Health.

[23] Başer A., Sönmez Ö.F., Kürklü Arpaçay D., Şahin H. (2025). Adapting organizational culture scale into healthcare professional education: A scale validity and reliability analysis. Hum. Resour. Health.

[24] Singh A., Purohit B.M., Bhambal A., Saxena S., Singh A., Gupta A. (2011). Knowledge, attitudes, and practice regarding infection control measures among dental students in Central India. J. Dent. Educ..

[25] Kishore J., Goel P., Sagar B., Joshi T.K. (2000). Awareness about biomedical waste management and infection control among dentists of a teaching hospital in New Delhi, India. Indian J. Dent. Res. Off. Publ. Indian Soc. Dent. Res..

[26] Moradi Khanghahi B., Jamali Z., Pournaghi Azar F., Naghavi Behzad M., Azami-Aghdash S. (2013). Knowledge, Attitude, Practice, and Status of Infection Control among Iranian Dentists and Dental Students: A Systematic Review. J. Dent. Res..

[27] Alkadi L., Farook F.F., Binmoghaiseeb I., Alyousef Y., Alabdulwahab A., Aljohani R., Asiri A. (2024). Knowledge and Compliance with Infection Prevention and Control Practices in Prosthodontic Procedures Among Dental Students and Professionals. Healthcare.

[28] Zia N., Doss J.G., Danaee M., John J., Panezai J. (2025). Psychometric analysis of a KAP questionnaire on green dentistry using PLS-SEM and EFA: A pilot study. BMC Oral Health.

[29] Coltman T., Devinney T.M., Midgley D.F., Venaik S. (2008). Formative versus reflective measurement models: Two applications of formative measurement. J. Bus. Res..

[30] Nassar M., Shalan W., Al-Janaby U., Elnagar H., Alawadhi M., Jaser S., Joury E. (2024). Exploring environmental sustainability in dentistry among students and educators in the United Arab Emirates: A cross-sectional survey. BMC Med. Educ..

[31] Bamedhaf O., Salman H., Tegginmani S.A., Guraya S.S. (2025). Environmental sustainability in the dental curriculum: A scoping review. BMC Med. Educ..

[32] Jalbani N., Doss J.G., John J., Panezai J. (2023). Sustainability in Dentistry: Assessing knowledge, attitude, and practices of dental practitioners about green dentistry. Pak. J. Med. Sci..

[33] Thakar S., Kinariwala N., Pandya D., Parekh N.H., Patel N.K., Patel A. (2023). Awareness and Constraints towards the Implementation of Green Dentistry amongst Dental Students and Private Practitioners of West India. J. Pharm. Bioallied Sci..

[34] Picard G., Prakash T., Labrèche F., Gravel S. (2025). Occupational exposures to chemicals in dentistry: A scoping review. J. Occup. Environ. Hyg..

[35] Boateng G.O., Neilands T.B., Frongillo E.A., Melgar-Quiñonez H.R., Young S.L. (2018). Best Practices for Developing and Validating Scales for Health, Social, and Behavioral Research: A Primer. Front. Public Health.

[36] Podsakoff P.M., MacKenzie S.B., Lee J.Y., Podsakoff N.P. (2003). Common method biases in behavioral research: A critical review of the literature and recommended remedies. J. Appl. Psychol..

[37] Diamantopoulos A., Winklhofer H.M. (2001). Index Construction with Formative Indicators: An Alternative to Scale Development. J. Mark. Res..

[38] Fabrigar L.R., Wegener D.T., MacCallum R.C., Strahan E.J. (1999). Evaluating the use of exploratory factor analysis in psychological research. Psychol. Methods.

[39] Hair J.F., Hult G.T.M., Ringle C.M., Sarstedt M., Danks N.P., Ray S. (2021). Partial Least Squares Structural Equation Modeling (PLS-SEM) Using R: A Workbook [Internet].

[40] Mokkink L.B., Elsman E.B.M., Terwee C.B. (2024). COSMIN guideline for systematic reviews of patient-reported outcome measures version 2.0. Qual. Life Res..

[41] Mokkink L.B., Boers M., Van Der Vleuten C.P.M., Bouter L.M., Alonso J., Patrick D.L., de Vet H.C.W., Terwee C.B. (2020). COSMIN Risk of Bias tool to assess the quality of studies on reliability or measurement error of outcome measurement instruments: A Delphi study. BMC Med. Res. Methodol..

[42] Mokkink L.B., Terwee C.B., Patrick D.L., Alonso J., Stratford P.W., Knol D.L., Bouter L.M., de Vet H.C.W. (2010). The COSMIN checklist for assessing the methodological quality of studies on measurement properties of health status measurement instruments: An international Delphi study. Qual. Life Res..

[43] Shi D., Lee T., Maydeu-Olivares A. (2019). Understanding the model size effect on SEM fit indices. Educ. Psychol. Meas..

[44] Bandura A. (2001). Social Cognitive Theory: An Agentic Perspective. Annu. Rev. Psychol..

[45] Fisal A.A., Rohani M.M., Aziz A.A., Hussain H.H., Fuad M.A.A. (2026). Knowledge, Awareness, and Practices of Green Dentistry Among Dental Undergraduate Students: A Cross-Sectional Study. J. Dent. Educ..

[46] Sönmez Ö.F., Başer A., Sofuoğlu Z. (2025). Climate Change Perceptions Scale for Health and Related Professionals and Students (CCPS-HARPS)—Development, reliability and validity. J. Clim. Change Health.

[47] Moussa L.G., Sonmez O.F., Bedi R. (2025). Incorporating oral health into the One Health agenda: Contributions and implications. Sci. One Health.

[48] Duane B., Ramasubbu D., Harford S., Steinbach I., Stancliffe R., Ballantyne G. (2019). Environmental sustainability and biodiversity within the dental practice. Br. Dent. J..

[49] Jamal H., Marghalani A.A., Al-Sharif A., Shinawi A., Gaffar B., Al-Edaili E.A., Al-Baqami G., AlQarni M. (2023). Exploring the Perception of Dental Undergraduate Students and Faculty on Environmental Sustainability in Dentistry: A Cross-Sectional Survey in 26 Dental Schools in Saudi Arabia. Dent. J..

[50] Mahdi S.S., Ahmed Z., Allana R., Amenta F., Agha D., Latif M.W., Daood U., Mehanna C. (2021). Knowledge, Attitudes, and Perceptions of Dental Assistants regarding Dental Asepsis and Sterilization in the Dental Workplace. Int. J. Dent..

[51] Mai F., Parkinson E., Mumford F., Shehabi Z. (2026). Interactive workshops on improving dental students’ understanding of environmentally sustainable dentistry: A quality improvement project. BDJ Open.

[52] Dixon J., Baird H.M., Field J., Martin N. (2025). Longitudinal integration of environmental sustainability in the dental curriculum: Assessing changes in student awareness, attitudes and knowledge. J. Dent..

[53] Sönmez Ö.F., Prenga G., Başer A., Belli S., Çelik G. (2026). Strengthening climate-health literacy through sustainability education among dental students: A quasi-experimental evaluation. BMC Med. Educ..

