# Supplementary material for: Testing and Validating Green Dental Laboratory ‘Knowledge, Attitude, and Practice’ (KAP) Scale Developed for Dental Technicians Doing Prosthodontic Work
Source: Healthcare (Basel). 2026 Oct 12;14(20):3399. doi: 10.3390/healthcare14203399 (PMC13704541; doi:10.3390/healthcare14203399)
Supplement: Supplementary file 1 [file healthcare-14-03399-s001.zip › healthcare-4510310-supplementary.pdf]

SUPPLEMENTARY MATERIAL

Table S1. Item-Level Descriptive Statistics (N = 194).

Descriptive statistics for all 49 items of the Green Dental Laboratory KAP Scale

Construct	Item	n	Mean	SD	% Yes	Skewness	Kurtosis	Corrected item–total r
**Knowledge**	B1	191	0.72	0.45	72.3	-1.00	-1.01	0.571
**Knowledge**	B2	193	0.33	0.47	33.2	0.72	-1.50	0.400
**Knowledge**	B3	193	0.58	0.50	57.5	-0.31	-1.93	0.482
**Knowledge**	B4	193	0.63	0.48	63.2	-0.55	-1.71	0.529
**Knowledge**	B5	193	0.63	0.48	62.7	-0.53	-1.74	0.526
**Knowledge**	B6	192	0.41	0.49	40.6	0.38	-1.87	0.565
**Knowledge**	B7	192	0.35	0.48	34.9	0.64	-1.61	0.487
**Knowledge**	B8	191	0.48	0.50	48.2	0.07	-2.02	0.524
**Knowledge**	B9	192	0.60	0.49	60.4	-0.43	-1.83	0.438
**Knowledge**	B10	188	0.43	0.50	42.6	0.30	-1.93	0.557
**Knowledge**	B11	194	0.75	0.44	74.7	-1.15	-0.69	0.459
**Attitude**	T1	194	3.78	1.34	—	-0.85	-0.49	0.881
**Attitude**	T2	194	3.61	1.31	—	-0.60	-0.73	0.827
**Attitude**	T3	194	3.70	1.34	—	-0.81	-0.53	0.871
**Attitude**	T4	194	3.81	1.34	—	-0.91	-0.40	0.877
**Attitude**	T5	194	3.94	1.34	—	-1.06	-0.19	0.889
**Attitude**	T6	194	3.77	1.27	—	-0.82	-0.39	0.894
**Attitude**	T7	194	3.76	1.25	—	-0.77	-0.45	0.890
**Attitude**	T8	194	3.74	1.36	—	-0.79	-0.56	0.832
**Attitude**	T9	194	3.75	1.30	—	-0.71	-0.60	0.816
**Attitude**	T10	194	3.88	1.37	—	-1.00	-0.26	0.899
**Attitude**	T11	194	3.85	1.28	—	-0.92	-0.23	0.890
**Attitude**	T12	194	2.77	1.40	—	0.27	-1.13	0.159
**Attitude**	T13	194	3.21	1.24	—	-0.20	-0.81	0.438
**Attitude**	T14	194	2.65	1.38	—	0.33	-1.08	0.109
**Attitude**	T15	194	2.99	1.15	—	0.15	-0.61	0.228
**Attitude**	T16	194	3.66	1.31	—	-0.69	-0.62	0.821
**Attitude**	T17	194	3.62	1.32	—	-0.63	-0.70	0.856
**Attitude**	T18	194	3.68	1.27	—	-0.68	-0.53	0.863

**Attitude**	T19	194	3.72	1.25	—	-0.70	-0.44	0.864
**Practice**	U1	194	2.70	1.34	—	0.21	-1.03	0.590
**Practice**	U2	194	2.59	1.36	—	0.28	-1.12	0.653
**Practice**	U3	194	2.88	1.40	—	0.10	-1.18	0.755
**Practice**	U4	194	2.75	1.39	—	0.21	-1.15	0.707
**Practice**	U5	194	2.91	1.38	—	0.04	-1.16	0.668
**Practice**	U6	194	2.97	1.44	—	-0.03	-1.29	0.742
**Practice**	U7	194	3.75	1.36	—	-0.73	-0.63	0.579
**Practice**	U8	194	3.39	1.30	—	-0.36	-0.90	0.670
**Practice**	U9	194	3.43	1.24	—	-0.42	-0.71	0.683
**Practice**	U10	194	3.24	1.39	—	-0.23	-1.11	0.746
**Practice**	U11	194	2.73	1.43	—	0.19	-1.22	0.637
**Practice**	U12	194	3.23	1.35	—	-0.27	-0.97	0.630
**Practice**	U13	194	3.16	1.37	—	-0.18	-1.08	0.715
**Practice**	U14	194	3.22	1.38	—	-0.24	-1.07	0.599
**Practice**	U15	194	3.26	1.34	—	-0.24	-1.05	0.741
**Practice**	U16	194	3.11	1.42	—	-0.14	-1.22	0.794
**Practice**	U17	194	3.19	1.39	—	-0.19	-1.14	0.713
**Practice**	U18	194	3.15	1.34	—	-0.21	-1.02	0.805
**Practice**	U19	194	3.07	1.34	—	-0.13	-1.03	0.804

Knowledge items (B1–B11) were scored dichotomously (Yes = 1, No = 0); % Yes is reported in place of a mean interpretation. Attitude (T1–T19) and Practice (U1–U19) items were scored on a 5-point Likert scale. Kurtosis values are excess kurtosis (normal distribution = 0). Corrected item–total correlation is the Pearson correlation between each item and the sum of the remaining items within its own construct (all 19 attitude items included, prior to exclusion of T12–T15 for factor analysis). Missing values (present only for a small number of Knowledge items, 1–6 cases per item) were excluded pairwise for this table.

Table S2. Exploratory Factor Analysis of the Attitude Construct.

Principal Axis Factoring, unrotated single-factor solution (n = 194; 15 retained items)

Item	Factor loading	Communality (h^2)
**T1**	0.927	0.859
**T2**	0.869	0.755
**T3**	0.906	0.821
**T4**	0.910	0.829
**T5**	0.917	0.841
**T6**	0.921	0.848
**T7**	0.914	0.836
**T8**	0.864	0.747
**T9**	0.835	0.698
**T10**	0.934	0.872
**T11**	0.924	0.853
**T16**	0.853	0.728
**T17**	0.897	0.805
**T18**	0.905	0.819
**T19**	0.909	0.827

Extraction method: Principal Axis Factoring (iterative, initial communality estimates = squared multiple correlations), consistent with the method reported in the main text. Kaiser–Meyer–Olkin measure of sampling adequacy = 0.967; Bartlett's test of sphericity $\chi 2(105) = 4391.501$, $p < .001$. Initial eigenvalue for the first (retained) factor = 12.326, accounting for 82.17% of total variance, consistent with the single-factor solution reported in the main text and Figure 1 (scree plot). Loadings and communalities shown here are the Extraction-phase values from the iterated Principal Axis solution. Four negatively worded items (T12–T15) were excluded from this analysis.

Table S3. Confirmatory Factor Analysis of the Attitude Construct.

Single-factor CFA, maximum-likelihood estimation (n = 194; 15 retained items: T1–T11, T16–T19)

Item	Unstandardized loading	Standardized loading	SE	z	p
**T1**	1.000	0.927	— [a]	— [a]	— [a]
**T2**	0.913	0.870	0.046	19.88	<0.001
**T3**	0.975	0.907	0.043	22.48	<0.001
**T4**	0.982	0.912	0.043	22.88	<0.001
**T5**	0.990	0.920	0.042	23.56	<0.001
**T6**	0.942	0.923	0.040	23.76	<0.001
**T7**	0.921	0.917	0.040	23.25	<0.001
**T8**	0.938	0.862	0.048	19.39	<0.001
**T9**	0.865	0.832	0.049	17.75	<0.001
**T10**	1.021	0.931	0.042	24.58	<0.001
**T11**	0.951	0.923	0.040	23.78	<0.001
**T16**	0.898	0.851	0.048	18.76	<0.001
**T17**	0.945	0.896	0.044	21.63	<0.001
**T18**	0.919	0.903	0.041	22.15	<0.001
**T19**	0.912	0.908	0.041	22.51	<0.001

[a] *T1 was fixed as the reference (marker) indicator (unstandardized loading = 1.0) for model identification; standard error, z, and p-value are not estimated for a fixed parameter, and its standardized loading is a derived (not freely estimated) value.*

Model fit indices

χ^2	df	χ^2/df	CFI	TLI	SRMR	RMSEA	RMSEA 90% CI
**459.851**	90	5.11	0.917	0.903	0.030	0.146	0.133–0.159

Fit indices computed via maximum-likelihood (ML) estimation in Python (semopy), reproducing the main-text values (χ2(90) = 459.851, CFI = 0.917, TLI = 0.903, SRMR = 0.030, RMSEA = 0.146) to three decimal places. The main text additionally reports a robust (MLR/Satorra–Bentler-corrected) RMSEA of 0.130 from the original lavaan analysis; the robust correction is not available in the software used to generate this table, so the uncorrected ML-based RMSEA and its 90% confidence interval (computed from the noncentral χ2 distribution) are shown here. Standardized loadings ranged from 0.832 (T9) to 0.931 (T10), consistent with the 0.83–0.93 range reported in the main text.

Table S4. Formative Indicator Collinearity Statistics (VIF).

Variance Inflation Factors for the Knowledge (B1–B11) and Practice (U1–U19) formative constructs

Construct	Item	VIF
**Knowledge**	B1	1.721
**Knowledge**	B2	1.230
**Knowledge**	B3	1.439
**Knowledge**	B4	1.501
**Knowledge**	B5	1.498
**Knowledge**	B6	1.565
**Knowledge**	B7	1.424
**Knowledge**	B8	1.505
**Knowledge**	B9	1.366
**Knowledge**	B10	1.566
**Knowledge**	B11	1.351
**Practice**	U1	2.383
**Practice**	U2	2.770
**Practice**	U3	3.030
**Practice**	U4	3.274
**Practice**	U5	2.665
**Practice**	U6	2.731
**Practice**	U7	2.096
**Practice**	U8	2.398
**Practice**	U9	2.614
**Practice**	U10	3.148
**Practice**	U11	1.957
**Practice**	U12	2.139
**Practice**	U13	2.507
**Practice**	U14	1.909
**Practice**	U15	2.808
**Practice**	U16	3.631
**Practice**	U17	2.512
**Practice**	U18	4.663
**Practice**	U19	4.312

VIF for each indicator was computed by regressing it on the remaining indicators within the same formative block, following the standard formative-measurement diagnostic procedure. Knowledge VIF ranged from 1.230 (B2) to 1.721 (B1); Practice VIF ranged from 1.909 (U14) to 4.663 (U18) — consistent with the ranges reported in the main text

Table S5. Cross Loadings/ Correlation Matrix.

Correlation among the Knowledge, Attitude, and Practice constructs (n = 194).

r	**Knowledge**	**Attitude**	**Practice**
**Knowledge**	1	.267**	.580**
**Attitude**	.267**	1	.510**
**Practice**	.580**	.510**	1

**. Correlation is significant at the 0.01 level (2-tailed).

Pearson correlation coefficients (*r*) ranged from 0.267 (Knowledge-Attitude) to 0.580 (Knowledge-Practice), with all inter-construct correlations remaining statistically significant ($p<0.001$). All correlation values are well below the critical threshold of 0.85, confirming that while the constructs are conceptually related, they maintain sufficient empirical difference without any multicollinearity issues.

Table S6. Formative Indicator Measurement Model Assessment, Green Dental Laboratory Knowledge, Attitude, and Practice (KAP) Scale and COSMIN Assessment.

Construct	Item	Outer weight	SE	T-statistic	p-value	Outer loading	VIF	Decision (Justification)
**Knowledge**	B1	0.142	0.068	2.088	**0.038**	0.68	1.721	Retained (p<0.05, loading>0.50)
	B2	0.185	0.062	2.984	**0.003**	0.48	1.230	Retained (p<0.001, only one with loading <0.50)
	B3	0.089	0.071	1.254	0.211	0.55	1.439	Retained (loading>0.50)
	B4	0.210	0.059	3.559	**< 0.001**	0.64	1.501	Retained (p<0.05, loading>0.50)
	B5	0.164	0.065	2.523	**0.012**	0.62	1.498	Retained (p<0.05, loading>0.50)
	B6	0.074	0.069	1.072	0.285	0.66	1.565	Retained (loading>0.50)
	B7	0.198	0.061	3.246	**0.001**	0.58	1.424	Retained (p<0.05, loading>0.50)
	B8	0.131	0.067	1.955	0.052	0.61	1.505	Retained (loading>0.50)
	B9	0.155	0.064	2.422	**0.016**	0.52	1.366	Retained (p<0.05, loading>0.50)
	B10	0.062	0.070	0.886	0.377	0.65	1.566	Retained (loading>0.50)
	B11	0.176	0.063	2.794	**0.006**	0.72	1.351	Retained (p<0.05, loading>0.50)
**Practice**	U1	0.092	0.041	2.244	**0.026**	0.65	2.383	Retained (p<0.05, loading>0.50)
	U2	0.115	0.038	3.026	**0.003**	0.70	2.770	Retained (p<0.05, loading>0.50)
	U3	0.041	0.045	0.911	0.363	0.78	3.030	Retained (loading>0.50)
	U4	0.138	0.036	3.833	**< 0.001**	0.74	3.274	Retained (p<0.05, loading>0.50)
	U5	0.085	0.042	2.024	**0.044**	0.71	2.665	Retained (p<0.05, loading>0.50)
	U6	0.104	0.039	2.667	**0.008**	0.77	2.731	Retained (p<0.05, loading>0.50)
	U7	0.032	0.046	0.696	0.487	0.62	2.096	Retained (loading>0.50)
	U8	0.126	0.037	3.405	**0.001**	0.70	2.398	Retained (p<0.05, loading>0.50)
	U9	0.078	0.043	1.814	0.071	0.72	2.614	Retained (loading>0.50)
	U10	0.145	0.035	4.143	**< 0.001**	0.78	3.148	Retained (p<0.05, loading>0.50)
	U11	0.099	0.040	2.475	0.014	0.67	1.957	Retained (loading>0.50)
	U12	0.162	0.034	4.765	**< 0.001**	0.66	2.139	Retained (p<0.05, loading>0.50)
	U13	0.181	0.033	5.485	**< 0.001**	0.75	2.507	Retained (p<0.05, loading>0.50)
	U14	0.194	0.032	6.063	**< 0.001**	0.64	1.909	Retained (p<0.05, loading>0.50)
	U15	0.150	0.035	4.286	**< 0.001**	0.77	2.808	Retained (p<0.05, loading>0.50)
	U16	0.088	0.042	2.095	**0.037**	0.82	3.631	Retained (p<0.05, loading>0.50)
	U17	0.051	0.044	1.159	0.248	0.74	2.512	Retained (loading>0.50)
	U18	0.110	0.039	2.821	**0.005**	0.83	4.663	Retained (p<0.05, loading>0.50)
	U19	0.121	0.038	3.184	**0.002**	0.83	4.312	Retained (p<0.05, loading>0.50)

Yeşil Diş Laboratuvarı Bilgi, Tutum ve Uygulama (KAP) Ölçeği

Green Dental Laboratory Knowledge, Attitude, and Practice (KAP) Scale, Administered Version and Validated Analytical Core, Turkish original with English translation

Item wording is presented in Turkish, as administered, with the English translation given in parentheses. The questionnaire administered to participants comprised 49 items (11 knowledge, 19 attitude, 19 practice items). During psychometric evaluation, four reverse-worded Attitude items (T12–T15) were excluded from the factor and reliability analyses. Accordingly, the validated analytical core comprises 45 items: 11 Knowledge items (B1–B11), 15 Attitude items (T1–T11 and T16–T19), and 19 Practice items (U1–U19). T12–T15 are retained in this Supplementary Material to transparently document the questionnaire as administered but are not part of the validated Attitude score and should not be included in its calculation or psychometric interpretation.

Bölüm A: Demografik Bilgiler / Section A: Demographic Information

Variable (Turkish)	Variable (English)	Response options
Cinsiyet	Gender	Kadın / Erkek (Female / Male)
Yaş	Age	18-24, 25-34, 35-44, 45-54, 55 ve üzeri (55 and above)
Eğitim Durumu	Education level	Ortaöğretim, Ön lisans, Lisans, Lisansüstü (Secondary, Associate, Bachelor's, Postgraduate)
Mesleki Deneyim	Professional experience	<1, 1-5, 6-10, 11-20, >20 yıl (years)
Çalıştığı Laboratuvar Türü	Type of laboratory	Özel, Kamu/Hastane, Üniversite, Diğer (Private, Public/Hospital, University, Other)
Çalışan Sayısı	Number of laboratory staff	1-5, 6-10, 11-20, >20
'Yeşil Laboratuvar' kavramını daha önce duydunuz mu?	Have you heard of the “Green Laboratory” concept before?	Evet / Hayır (Yes / No)
Çevresel sürdürülebilirlik eğitimi aldınız mı?	Have you received environmental sustainability training?	Evet / Hayır (Yes / No)

Bölüm B: Bilgi / Section B: Knowledge (B1-B11)

Item	Wording (Turkish, as administered; English translation)	Response options
B1	Diş laboratuvarı atıklarının bir bölümünün tehlikeli atık sınıfında yer aldığını ve özel lisanslı firmalar aracılığıyla bertaraf edilmesi gerektiğini biliyor musunuz? *(Do you know that some dental laboratory waste is classified as hazardous waste and must be disposed of through specially licensed companies?)*	Evet=1 / Hayır=0 (Yes=1 / No=0)
B2	Diş laboratuvarlarında 'sürdürülebilir (yeşil) uygulama' kavramının anlamını bildiğinizi düşünüyor musunuz? *(Do you think you know the meaning of the concept of "sustainable (green) practice" in dental laboratories?)*	Evet=1 / Hayır=0 (Yes=1 / No=0)
B3	Dijital iş akışının (CAD/CAM + intraoral tarayıcı) geleneksel yöntemlere kıyasla toplam karbon ayak izini azaltabileceğine dair bilimsel kanıtların mevcut olduğunu biliyor musunuz? *(Do you know that there is scientific evidence that digital workflows (CAD/CAM plus intraoral scanning) can reduce the total carbon footprint compared with conventional methods?)*	Evet=1 / Hayır=0 (Yes=1 / No=0)
B4	Metal alaşım döküm atıklarının (nikel, kobalt-krom, titanyum vb.) özel bertaraf gerektirdiğini biliyor musunuz? *(Do you know that metal alloy casting waste (nickel, cobalt-chromium, titanium, etc.) requires special disposal?)*	Evet=1 / Hayır=0 (Yes=1 / No=0)
B5	Monomer ve akrilik toz gibi kimyasal atıkların kanalizasyona dökülmesinin yasak olduğunu biliyor musunuz? *(Do you know that it is prohibited to dispose of chemical waste such as monomer and acrylic powder into the sewage system?)*	Evet=1 / Hayır=0 (Yes=1 / No=0)
B6	Kullanılmış alçı, silikon ve polivinil siloksan (PVS) ölçü materyallerinin geri dönüşüm programları bulunduğunu biliyor musunuz? *(Do you know that recycling programs exist for used gypsum, silicone, and polyvinyl siloxane (PVS) impression materials?)*	Evet=1 / Hayır=0 (Yes=1 / No=0)
B7	Diş laboratuvarlarında oluşan ince toz partikülleri (PM2.5) için sınır değerlerin belirlenmiş olduğunu biliyor musunuz? *(Do you know that exposure limit values have been established for fine particulate matter (PM2.5) generated in dental laboratories?)*	Evet=1 / Hayır=0 (Yes=1 / No=0)
B8	Poliüretan ve bazı baskı reçinelerinin (3D baskı) bertarafında özel düzenlemelerin gerektiğini biliyor musunuz? *(Do you know that the disposal of polyurethane and certain 3D-printing resins requires special regulations?)*	Evet=1 / Hayır=0 (Yes=1 / No=0)
B9	Porselen/seramik fırınlarının enerji tüketiminin çevresel etkisinin farkında mısınız? *(Are you aware of the environmental impact of the energy consumption of porcelain/ceramic furnaces?)*	Evet=1 / Hayır=0 (Yes=1 / No=0)
B10	Diş laboratuvarlarında su tasarrufu için kullanılabilecek sistemleri (ör. kapalı devre soğutma) biliyor musunuz? *(Do you know about systems that can be used for water conservation in dental laboratories, such as closed-circuit cooling?)*	Evet=1 / Hayır=0 (Yes=1 / No=0)
B11	Enerji tasarruflu ekipman seçiminin (A++ sınıfı fırınlar, LED aydınlatma vb.) laboratuvar karbon ayak izini azaltabileceğini biliyor musunuz? *(Do you know that choosing energy-efficient equipment (A++ class furnaces, LED lighting, etc.) can reduce the laboratory's carbon footprint?)*	Evet=1 / Hayır=0 (Yes=1 / No=0)

Bölüm C: Tutum / Section C: Attitude (T1-T19)

Item	Wording (Turkish, as administered; English translation)	Response options
T1	Diş laboratuvarlarının çevresel etkilerini azaltması mesleki bir sorumluluktur. *(Reducing the environmental impact of dental laboratories is a professional responsibility.)*	1-5 (1=Kesinlikle Katılmıyorum/Strongly Disagree, 5=Kesinlikle Katılıyorum/Strongly Agree)
T2	Bir diş teknisyeni olarak çalışma süreçlerimin çevre üzerindeki etkilerinden kişisel olarak sorumlu olduğumu düşünürüm. *(As a dental technician, I feel personally responsible for the environmental impact of my work processes.)*	1-5 (1=Kesinlikle Katılmıyorum/Strongly Disagree, 5=Kesinlikle Katılıyorum/Strongly Agree)
T3	Çevresel sürdürülebilirlik, laboratuvar hizmet kalitesinin bir parçası olarak değerlendirilmelidir. *(Environmental sustainability should be considered part of laboratory service quality.)*	1-5 (1=Kesinlikle Katılmıyorum/Strongly Disagree, 5=Kesinlikle Katılıyorum/Strongly Agree)
T4	Metal alaşım atıklarının kontrolsüz şekilde uzaklaştırılması çevre açısından ciddi bir sorundur. *(Uncontrolled disposal of metal alloy waste is a serious environmental problem.)*	1-5 (1=Kesinlikle Katılmıyorum/Strongly Disagree, 5=Kesinlikle Katılıyorum/Strongly Agree)
T5	Kimyasal atıkların uygun şekilde ayrıştırılması, laboratuvar güvenliği kadar çevre sağlığı açısından da önemlidir. *(Proper separation of chemical waste is important for environmental health as well as laboratory safety.)*	1-5 (1=Kesinlikle Katılmıyorum/Strongly Disagree, 5=Kesinlikle Katılıyorum/Strongly Agree)
T6	Reçine, monomer ve benzeri maddelerin ayrı toplanması gereksiz değil, gerekli bir uygulamadır. *(Separately collecting resin, monomer, and similar substances is a necessary practice, not an unnecessary one.)*	1-5 (1=Kesinlikle Katılmıyorum/Strongly Disagree, 5=Kesinlikle Katılıyorum/Strongly Agree)
T7	Laboratuvar atıklarının türüne göre ayrılması ek iş yükü oluştursa bile sürdürülmesi gereken bir uygulamadır. *(Sorting laboratory waste by type is a practice that should be maintained even if it creates additional workload.)*	1-5 (1=Kesinlikle Katılmıyorum/Strongly Disagree, 5=Kesinlikle Katılıyorum/Strongly Agree)
T8	Çevresel yarar sağlayan dijital teknolojilere geçiş yapmaya istekliyim. *(I am willing to transition to digital technologies that provide environmental benefits.)*	1-5 (1=Kesinlikle Katılmıyorum/Strongly Disagree, 5=Kesinlikle Katılıyorum/Strongly Agree)

T9	CAD/CAM ve dijital iş akışı, uygun koşullarda geleneksel yöntemlere göre daha sürdürülebilir bir seçenek olabilir. *(Under appropriate conditions, CAD/CAM and digital workflows can be a more sustainable option than conventional methods.)*	1-5 (1=Kesinlikle Katılmıyorum/Strongly Disagree, 5=Kesinlikle Katılıyorum/Strongly Agree)
T10	Yeni çevre dostu materyal ve teknolojileri öğrenmek ve denemek, mesleki gelişimin bir parçasıdır. *(Learning and trying new environmentally friendly materials and technologies is part of professional development.)*	1-5 (1=Kesinlikle Katılmıyorum/Strongly Disagree, 5=Kesinlikle Katılıyorum/Strongly Agree)
T11	Çevresel yarar sağlayan yeni uygulamalar başlangıçta ek çaba gerektirse de desteklenmelidir. *(New practices that provide environmental benefits should be supported even if they require extra effort initially.)*	1-5 (1=Kesinlikle Katılmıyorum/Strongly Disagree, 5=Kesinlikle Katılıyorum/Strongly Agree)
T12*	Çevre dostu uygulamalar laboratuvarlar için çoğu zaman gereksiz bir mali yük oluşturur. *(Environmentally friendly practices often create an unnecessary financial burden for laboratories.)*	1-5 (1=Kesinlikle Katılmıyorum/Strongly Disagree, 5=Kesinlikle Katılıyorum/Strongly Agree)
T13*	Küçük ölçekli laboratuvarlarda çevre dostu uygulamaları sürdürmek pratikte zordur. *(Maintaining environmentally friendly practices is practically difficult in small-scale laboratories.)*	1-5 (1=Kesinlikle Katılmıyorum/Strongly Disagree, 5=Kesinlikle Katılıyorum/Strongly Agree)
T14*	Çevresel sürdürülebilirlik uygulamaları ancak ekonomik açıdan doğrudan kazanç sağlıyorsa uygulanmalıdır. *(Environmental sustainability practices should only be implemented if they provide a direct economic gain.)*	1-5 (1=Kesinlikle Katılmıyorum/Strongly Disagree, 5=Kesinlikle Katılıyorum/Strongly Agree)
T15*	Çevresel standartlar ve sertifikasyon süreçleri, laboratuvarlar için çoğu zaman gereğinden fazla bürokrasi yaratır. *(Environmental standards and certification processes often create excessive bureaucracy for laboratories.)*	1-5 (1=Kesinlikle Katılmıyorum/Strongly Disagree, 5=Kesinlikle Katılıyorum/Strongly Agree)
T16	Diş teknisyeni eğitiminde çevresel sürdürülebilirlik konularına mutlaka yer verilmelidir. *(Environmental sustainability topics should definitely be included in dental technician education.)*	1-5 (1=Kesinlikle Katılmıyorum/Strongly Disagree, 5=Kesinlikle Katılıyorum/Strongly Agree)
T17	Mezuniyet sonrası eğitimlerde yeşil laboratuvar uygulamalarına ilişkin içerikler artırılmalıdır. *(Content related to green laboratory practices should be increased in post-graduation training.)*	1-5 (1=Kesinlikle Katılmıyorum/Strongly Disagree, 5=Kesinlikle Katılıyorum/Strongly Agree)

T18	Çevre dostu laboratuvar uygulamaları konusunda eğitim almak mesleki açıdan yararlı olur. *(Receiving training on environmentally friendly laboratory practices is professionally beneficial.)*	1-5 (1=Kesinlikle Katılmıyorum/Strongly Disagree, 5=Kesinlikle Katılıyorum/Strongly Agree)
T19	Laboratuvarlarda çevresel sürdürülebilirlik konusunda kurumsal farkındalığı artıracak çalışmalar yapılmalıdır. *(Efforts should be made to increase institutional awareness of environmental sustainability in laboratories.)*	1-5 (1=Kesinlikle Katılmıyorum/Strongly Disagree, 5=Kesinlikle Katılıyorum/Strongly Agree)

Bölüm D: Uygulama / Section D: Practice (U1-U19)

Item	Wording (Turkish, as administered; English translation)	Response options
U1	Çalıştığım laboratuvarda kullanılmış metal alaşımlarının lisanslı geri dönüşüm firmalarına teslim edilmesi sağlanır. *(In the laboratory where I work, used metal alloys are ensured to be handed over to licensed recycling companies.)*	1-5 (1=Hiçbir Zaman/Never, 5=Her Zaman/Always)
U2	Döküm atığı metalleri yeniden kullanım veya geri dönüşüm amacıyla biriktiriyorum. *(I collect casting waste metals for reuse or recycling purposes.)*	1-5 (1=Hiçbir Zaman/Never, 5=Her Zaman/Always)
U3	Mümkün olduğunda tek kullanımlık yerine yeniden kullanılabilir döküm sistemleri kullanıyorum. *(Whenever possible, I use reusable casting systems instead of single-use ones.)*	1-5 (1=Hiçbir Zaman/Never, 5=Her Zaman/Always)
U4	Monomer, asit ve çözücü atıklarını evsel atıklardan ayrı olarak, işaretli kaplarda biriktiriyorum. *(I collect monomer, acid, and solvent waste separately from household waste in labeled containers.)*	1-5 (1=Hiçbir Zaman/Never, 5=Her Zaman/Always)
U5	Alçı ve revetman atıklarını katı atık olarak ayrı topluyorum. *(I collect gypsum and investment material waste separately as solid waste.)*	1-5 (1=Hiçbir Zaman/Never, 5=Her Zaman/Always)
U6	3D baskı kullanılan durumlarda reçine ve destek materyali atıklarını uygun şekilde bertaraf ediyorum. *(When 3D printing is used, I dispose of resin and support material waste appropriately.)*	1-5 (1=Hiçbir Zaman/Never, 5=Her Zaman/Always)
U7	Kullanılmayan ekipmanları (fırınlar, polimerize cihazlar, kompresörler) çalışma saati dışında kapatıyorum. *(I turn off unused equipment (furnaces, polymerization devices, compressors) outside working hours.)*	1-5 (1=Hiçbir Zaman/Never, 5=Her Zaman/Always)
U8	Çalıştığım laboratuvarda enerji tasarruflu aydınlatma sistemleri kullanılmaktadır. *(Energy-efficient lighting systems are used in the laboratory where I work.)*	1-5 (1=Hiçbir Zaman/Never, 5=Her Zaman/Always)
U9	Fırın kullanımını enerji tüketimini azaltacak şekilde planlıyorum. *(I plan furnace use in a way that reduces energy consumption.)*	1-5 (1=Hiçbir Zaman/Never, 5=Her Zaman/Always)
U10	Alçı modellerin yıkanması sırasında su tüketimini azaltacak uygulamalar kullanıyorum. *(I use practices that reduce water consumption when washing gypsum models.)*	1-5 (1=Hiçbir Zaman/Never, 5=Her Zaman/Always)

U11	Çalıştığım laboratuvarda frezeleme/CAD-CAM soğutma suyunun yeniden kullanımını sağlayan bir sistem bulunmaktadır. *(In the laboratory where I work, there is a system that enables the reuse of milling/CAD-CAM cooling water.)*	1-5 (1=Hiçbir Zaman/Never, 5=Her Zaman/Always)
U12	İş emirleri ve hasta kayıtlarını dijital ortamda takip ediyorum (kağıt kullanımını azaltmak amacıyla). *(I keep work orders and patient records in digital format, in order to reduce paper use.)*	1-5 (1=Hiçbir Zaman/Never, 5=Her Zaman/Always)
U13	Mümkün olduğunda dijital ölçü/tarama (intraoral tarayıcı veya model tarayıcısı) kullanarak fiziksel ölçü maddesi tüketimini azaltıyorum. *(Whenever possible, I use digital impressions/scanning (intraoral or model scanners) to reduce the consumption of physical impression material.)*	1-5 (1=Hiçbir Zaman/Never, 5=Her Zaman/Always)
U14	Kağıt kullanımı gerektiğinde çift yüzlü baskı tercih ediyorum. *(When paper use is necessary, I prefer double-sided printing.)*	1-5 (1=Hiçbir Zaman/Never, 5=Her Zaman/Always)
U15	Mümkün olduğunda tek kullanımlık yerine yeniden kullanılabilir koruyucu ekipmanları tercih ediyorum. *(Whenever possible, I prefer reusable protective equipment over single-use equipment.)*	1-5 (1=Hiçbir Zaman/Never, 5=Her Zaman/Always)
U16	Sterilizasyon sonrası oluşan ambalaj atıklarını uygun atık ayrıştırma kurallarına göre yönetiyorum. *(I manage packaging waste generated after sterilization according to appropriate waste-separation rules.)*	1-5 (1=Hiçbir Zaman/Never, 5=Her Zaman/Always)
U17	Toz ve aerosolleri filtrelemek için hava filtresi/aspirasyon sistemi kullanıyorum. *(I use an air filter/aspiration system to filter dust and aerosols.)*	1-5 (1=Hiçbir Zaman/Never, 5=Her Zaman/Always)
U18	Malzeme seçimi veya satın alma sürecinde çevre dostu/biyouyumlu alternatifleri dikkate alıyorum. *(I take environmentally friendly/biocompatible alternatives into consideration during material selection or purchasing.)*	1-5 (1=Hiçbir Zaman/Never, 5=Her Zaman/Always)
U19	Tedarikçi seçiminde gereksiz ambalajı azaltan veya sürdürülebilir ambalaj kullanan firmalara öncelik verilir. *(Priority is given to suppliers that reduce unnecessary packaging or use sustainable packaging when selecting suppliers.)*	1-5 (1=Hiçbir Zaman/Never, 5=Her Zaman/Always)

Scale Scoring Protocol:

The three domains of the Green Dental Laboratory KAP Scale were scored separately, and no overall or global KAP score was calculated.
For the Knowledge domain, the 11 dichotomous items (B1–B11) were scored as 1 for a correct/affirmative response and 0 for an incorrect/negative response and summed to obtain a total Knowledge score ranging from 0 to 11, with higher scores indicating greater knowledge.
The Attitude score was based on the 15 items retained after psychometric evaluation (T1–T11 and T16–T19), each rated on a five-point Likert scale from 1 (strongly disagree) to 5 (strongly agree). The four reverse-worded items (T12–T15) were excluded from the validated analytical core and were not included in the Attitude score.
The Practice domain comprised all 19 items (U1–U19), rated on a five-point Likert scale from 1 (never) to 5 (always). Attitude and Practice were scored separately on their original 1–5 response metric. No composite score combining Knowledge, Attitude, and Practice was calculated because the three domains represent conceptually distinct constructs and were modeled separately in the psychometric analyses.

COSMIN Assessment

Green Dental Laboratory Knowledge, Attitude and Practice (KAP) Scale

**Instrument / version**	**Green Dental Laboratory KAP Scale (49-item pre-final version; 45-item analytic model after exclusion of T12–T15)**	**Completed by / date**	**08 July 2026**
**Target population**	Dental technicians / laboratory staff		
**Study design**	Completed scale development and psychometric evaluation study		
**Data collection period**	Not explicitly reported in the manuscript; data collected after ethics approval (16 April 2026, decision 2026.04.19)		

Use these tables by marking one rating cell per standard and entering the manuscript section, appendix, protocol page, analysis output, or expert-review evidence in the Evidence / notes column. The wording was written in past tense for use after the study had already been conducted. "Target participants" was used instead of "patients" because the instrument was intended for dental technicians/laboratory staff.

For this KAP instrument, Structural validity and Internal consistency are usually most relevant for the Attitude and Practice sections. The Knowledge section was dichotomous and should generally be evaluated with a scoring key, item difficulty/discrimination, and KR-20 or an equivalent dichotomous-score reliability estimate, unless a different measurement model was justified.

1. General recommendations

No	COSMIN standard phrased for a completed study	Very good	Adequate	Doubtful	Inadequate / N/A	Evidence / notes
**G1**	A clear research aim was provided, including the instrument name/version, the target population, and the measurement properties that were evaluated.	☒	☐	☐	☐	Aim, instrument name, target population, and evaluated properties stated in the final Introduction paragraph and Abstract (Background/Methods).
**G2**	The construct(s) measured by the instrument were clearly described.	☒	☐	☐	☐	Three constructs (knowledge, attitude, practice) defined in Introduction para 3 and Methods "Instrument Structure and Data Management" (Table 2), with formative/reflective specification.

**G3**	The development process of the instrument was clearly described, including the target population for whom it was developed.	☒	☐	☐	☐	Three-stage development described in Methods "Instrument Development" (item pool → expert panel → pilot, n=40; Figure 1, Table 1); target population defined in "Study Design, Setting and Participants".
**G4**	The origin of the construct was clear: a theory, conceptual framework, formative/reflective model, or clear rationale was provided.	☒	☐	☐	☐	KAP framework and behaviour-change rationale given in Introduction (refs 11–13); formative vs reflective measurement logic justified with refs 18, 19, 27; Bandura (30) invoked in Discussion.
**G5**	The structure of the instrument was clearly described, including the number of items, subscales/domains, instructions, response options, and scoring algorithm.	☐	☒	☐	☐	49 items, three domains, response formats, and measurement models given in Table 2; instructions and full item wording in Supplementary Material. A scoring algorithm (e.g., computation of domain scores) is not explicitly described in the manuscript.
**G6**	Existing evidence on the quality of the instrument was clearly described, or the absence of prior evidence was explicitly stated.	☒	☐	☐	☐	Absence of any prior validated instrument for dental laboratory technicians explicitly stated (Introduction, final paragraphs; refs 18, 24); the related Zia et al. pilot instrument is discussed.
**G7**	The context of use was clearly described, including intended application, setting, and administration mode.	☐	☒	☐	☐	Intended application (education, curriculum/workplace

						evaluation) described in "Implications for Education and Practice"; pilot administered online. The administration mode of the main data collection is not explicitly stated.
**G8**	Inclusion and exclusion criteria for the study participants were clearly described.	☐	☒	☐	☐	Eligible participants defined (students enrolled in Dental Prosthesis Technology programs or graduate technicians); duplicates and incomplete responses excluded. No formally itemized inclusion/exclusion criteria list.
**G9**	The method used to select participants was clearly described, such as convenience, consecutive, purposive, stratified, or random sampling.	☒	☐	☐	☐	Convenience sampling from Kırıkkale University, Vocational School of Health Services, explicitly stated and justified for preliminary validation ("Study Design, Setting and Participants").
**G10**	The extent to which the selected sample represented the intended target population was described using relevant participant and setting characteristics.	☒	☐	☐	☐	Table 3 reports gender, age, education, experience, laboratory type/size, prior awareness and training; Strengths and Limitations transparently notes the sample better represents trainees than established technicians.

2. Content validity

No	COSMIN standard phrased for a completed study	Very good	Adequate	Doubtful	Inadequate / N/A	Evidence / notes
**C1**	From the perspective of target participants, an appropriate method was used to assess item relevance, comprehensiveness of the instrument, and comprehensibility of instructions, items, response options, and recall period, if applicable.	☐	☒	☐	☐	Pilot administration to 40 dental technology students assessed item clarity and comprehensibility, and pilot item statistics guided rewording (Table 1). Relevance/comprehensiveness were not formally rated by target participants themselves.
**C2**	From the perspective of professionals/experts, an appropriate method was used to assess the relevance of each item for the construct of interest and the comprehensiveness of the instrument.	☐	☒	☐	☐	Five-expert panel assessed each item for relevance, clarity, and domain fit and removed duplicative/ambiguous items ("Instrument Development"). No quantitative content-validity index (e.g., CVI) reported.
**C3**	Professionals/experts from all relevant disciplines were included.	☐	☒	☐	☐	Panel comprised medical education, endodontics, and prosthodontics/dental technology academics. No practicing laboratory technician or environmental-health expert was included on the panel.
**C4**	Each item was evaluated by an appropriate number of target participants or professionals/experts; for example, at least 7 in qualitative work or at least 50 in quantitative survey-based content-validity assessment.	☐	☐	☒	☐	Five experts is below the commonly recommended minimum of 7; the 40 pilot participants evaluated comprehensibility only, not item relevance in a

						quantitative content-validity design.
**C5**	Skilled group moderators or interviewers were used when group meetings or interviews were conducted.	☐	☐	☐	☒	No group meetings or qualitative interviews were conducted; expert review and an online pilot were used instead.
**C6**	Group meetings or interviews were based on an appropriate topic guide or interview guide.	☐	☐	☐	☒	Not applicable
**C7**	Group meetings or interviews were recorded and transcribed verbatim when qualitative interviews or focus groups were conducted.	☐	☐	☐	☒	Not applicable
**C8**	An appropriate and clearly justified approach was used to analyse the content-validity data.	☐	☐	☒	☐	Item retention/removal decisions were based on expert judgement and pilot item statistics (difficulty/discrimination; Table 1), but the analytic approach to the expert data was not formalized (no CVI/kappa).
**C9**	At least two researchers were involved in the content-validity analysis, or another strategy was used to reduce single-reviewer bias.	☐	☒	☐	☐	Item decisions combined an independent five-member expert panel with pilot statistics reviewed by the research team, reducing single-reviewer bias; the exact number of researchers analysing content-validity data is not stated.

3. Structural validity

No	COSMIN standard phrased for a completed study	Very good	Adequate	Doubtful	Inadequate / N/A	Evidence / notes
**S1**	For classical test theory analyses, confirmatory factor analysis was performed, or exploratory common factor analysis was performed and justified for the development stage.	☒	☐	☐	☐	EFA (PAF) used to establish dimensionality at the development stage and CFA (lavaan, MLR) performed on the attitude construct ("Statistical Analysis"; Results EFA/CFA subsections; Supplementary Tables S2–S3).
**S2**	Clear information was provided on how the factor analysis was performed, including software, estimation method, assumption checks, rotation method when applicable, and model-fit criteria.	☒	☐	☐	☐	Software (R lavaan, SPSS v32, RStudio), estimator (MLR), assumption checks (KMO = 0.967; Bartlett $\chi^2(105) = 4391.501$), retention criteria (eigenvalue > 1, scree plot, loading ≥ 0.40) and fit criteria (CFI/TLI ≥ 0.90, SRMR ≤ 0.08, RMSEA ≤ 0.08–0.10) all reported.
**S3**	For IRT/Rasch analyses, the selected model fitted the research question and the scale structure.	☐	☐	☐	☒	Not applicable
**S4**	Clear information was provided on how the IRT or Rasch analysis was performed, including software, model, estimation method, assumption checks, and model-fit criteria.	☐	☐	☐	☒	Not applicable
**S5**	The structural-validity analysis was performed in an adequate sample size, taking expected missing values into account; for factor analysis, the sample size target and achieved participant-to-item ratio were reported.	☒	☐	☐	☐	Sample-size target (5–10 per item; ≥190 for factor analysis, ref 25) and achieved n = 194 reported; for the 15-item attitude CFA this equals ≈13 participants per item.
**S6**	The way missing items were handled in the structural-validity analysis was clearly described.	☐	☒	☐	☐	Duplicate and incomplete responses were excluded prior to analysis (complete-

						case approach); item-level missing-data handling within analyses is not described in further detail.

4. Internal consistency

No	COSMIN standard phrased for a completed study	Very good	Adequate	Doubtful	Inadequate / N/A	Evidence / notes
**I1**	Evidence was provided that each scale or subscale for which internal consistency was calculated was unidimensional.	☒	☐	☐	☐	Unidimensionality of the attitude scale supported by EFA (single factor, eigenvalue 12.326, 82.17% variance) and CFA (single-factor model; loadings 0.832–0.931; Supplementary Tables S2–S3).
**I2**	The internal-consistency analysis was performed in an adequate sample size, taking expected missing values into account.	☒	☐	☐	☐	Internal consistency computed on the full analytic sample (n = 194), exceeding common minimum requirements (e.g., ≥100).
**I3**	The way missing items were handled in the internal-consistency analysis was clearly described.	☐	☒	☐	☐	Same complete-case dataset as the structural analyses (duplicates/incomplete responses excluded); no further detail on item-level missingness.
**I4**	For continuous or ordinal summed scores, Cronbach's alpha and/or McDonald's omega was calculated for each unidimensional scale or subscale.	☒	☐	☐	☐	Cronbach's α = 0.984 and composite reliability = 0.984 reported for the unidimensional attitude scale (with AVE = 0.807); Discussion also notes that α

						> 0.95 may signal item redundancy.
**I5**	For dichotomous scores, Cronbach's alpha and/or KR-20 was calculated for each unidimensional scale or subscale.	☐	☐	☐	☒	KR-20/alpha not calculated for the dichotomous knowledge items because knowledge was explicitly modelled as a formative construct (refs 19, 27), for which internal-consistency indices are not appropriate; indicators evaluated via outer weights/loadings and VIF instead (Supplementary Table S4).
**I6**	For IRT-based scores, the standard error of theta or a reliability/separation coefficient was calculated for each unidimensional scale or subscale when applicable.	☐	☐	☐	☒	Not applicable
